# Processed meat intake and chronic disease morbidity and mortality: An overview of systematic reviews and meta-analyses

**DOI:** 10.1371/journal.pone.0223883

**Published:** 2019-10-17

**Authors:** Mina Nicole Händel, Isabel Cardoso, Katrine Marie Rasmussen, Jeanett Friis Rohde, Ramune Jacobsen, Sabrina Mai Nielsen, Robin Christensen, Berit Lilienthal Heitmann

**Affiliations:** 1 The Parker Institute, Bispebjerg and Frederiksberg Hospital, University of Copenhagen, Copenhagen, Denmark; 2 Department of Pharmacy, University of Copenhagen, Copenhagen, Denmark; 3 Research Unit of Rheumatology, Department of Clinical Research, University of Southern Denmark, Odense University Hospital, Odense, Denmark; 4 The Boden Institute of Obesity, Nutrition, Exercise & Eating Disorders, University of Sydney, Sydney, Australia; 5 Department of Public Health, Section for General Practice, University of Copenhagen, Copenhagen, Denmark; McMaster University, CANADA

## Abstract

Despite the nutritional value of meat, a large volume of reviews and meta-analyses suggests that processed meat intake is associated with an increased risk of chronic diseases. However, assessments of the quality of these published reviews internal validity are generally lacking. We systematically reviewed and assessed the quality alongside summarizing the results of previously published systematic reviews and meta-analyses that examined the association between processed meat intake and cancers, type II diabetes (T2D), and cardiovascular diseases (CVD). Reviews and meta-analyses published until May 2018 were identified through a systematic literature search in the databases MEDLINE and EMBASE, and reference lists of included reviews. The quality of the systematic reviews and meta-analyses was assessed using A Measurement Tool to Assess Systematic Reviews (AMSTAR). All eligible reviews had to comply with two quality requirements: providing sufficient information on quality assessment of the primary studies and a comprehensive search. The results were summarized for T2D, CVD, and each of the different cancer types. The certainty in the estimates of the individual outcomes was rated using the Grading of Recommendations, Assessment, Development and Evaluations (GRADE) method. In total, 22 systematic reviews were eligible and thus included in this review. More than 100 reviews were excluded because quality assessment of the primary studies had not been performed. The AMSTAR score of the included reviews ranged from 5 to 8 indicating moderate quality. Overall, the quality assessments of primary studies of the reviews are generally lacking; the scientific quality of the systematic reviews reporting positive associations between processed meat intake and risk of various cancers, T2D and CVD is moderate, and the results from case-control studies suggest more often a positive association than the results from cohort studies. The overall certainty in the evidence was very low across all individual outcomes, due to serious risk of bias and imprecision.

## Introduction

Chronic diseases such as cancers, type II diabetes (T2D) and cardiovascular diseases (CVD) are a substantial burden to society globally. According to World Health Organization (WHO), 36 million deaths per year, equivalent to 63% of all global deaths, can be attributed to these diseases and almost 40% of these deaths occur before the age of 70 years [1]. Modifiable risk factors for the development of chronic diseases include smoking, alcohol intake, physical activity, and body weight. In addition, dietary behaviors play a major role in the development of many chronic diseases [2]. Specifically, prudent dietary patterns, such as the Mediterranean diet, have been shown to be associated with reduced risk of several chronic diseases and mortality, as well as being beneficial for some of the risk factors for chronic diseases, such as hypertension [3–6]. Conversely, the typical “Western” diet has been associated with a higher risk of chronic diseases [7], although, at least for CVD, the evidence has been conflicting [8].

One of the main components of the “Western” diet is meat and meat products, which make a substantial contribution to the daily dietary intake of total energy, protein and fat, as well as important vitamins and minerals. A high consumption of processed meat products in relation to the risk of multiple chronic diseases has been studied extensively in reviews and meta-analyses, which have led to recommendations to moderate the consumption of preserved meat, such as sausages, salami, bacon and ham, for disease risk reduction worldwide [9–12]. However, the lack of quality assessment of the reviews providing the evidence base for these recommendations needs to be acknowledged [13–15]. Reviews that are not systematic, i.e. narrative reviews suffers from flaws, such as lack of reproducibility, lack of transparent methods, and a large degree of subjectivity that may be misleading in the conclusions made. Consequently, some authors have suggested placing constraints on the inclusion criteria of the reviews in relation to search strategy and quality assessment [16, 17], to ensure that the included reviews are ‘systematic’ and to guarantee a minimum level of methodological thoroughness.

The objective of this study was to provide a critical assessment of the available systematic reviews that examined the association between processed meat intake and the most common chronic diseases, i.e. different types of cancers (incidence and mortality), T2D (incidence), and CVD (incidence and mortality). As part of the assessment of the internal validity of the systematic reviews, we further aimed to explore potential reasons for heterogeneous results in meta-analyses by considering variations in factors such as study design and quality.

## Methods

This systematic review was performed in accordance with the recommendations of the Cochrane Collaboration and the Preferred Reporting Items for Systematic Reviews and Meta-Analyses (PRISMA) guidelines [18]. The study protocol was pre-specified and registered in advance of the literature search in the International Prospective Register of Systematic Reviews (PROSPERO) (CRD42017055272). No changes to the methods were made after commencement of this protocol, except for a *post hoc* quality evaluation of one review. This evaluation was performed using A Measurement Tool to Assess Systematic Reviews (AMSTAR).

One researcher (IC) conducted the literature search using a pre-specified search strategy (**S1 Table**), and two out of four researchers independently (MNH, IC, KMR, JFR) screened titles and abstracts of the selected articles, thoroughly assessed the full text reports according to the eligibility criteria, and performed data extraction of each of the studies eligible for inclusion. The review authors were not blinded to the journal titles, study authors/institutions or year of publication. The quality assessments using A Measurement Tool to Assess Systematic Reviews (AMSTAR) were completed independently by two out of four researchers (MNH, IC, KMR, JFR). Disagreements were resolved through discussions or by consulting a third reviewer (BLH).

### Search strategy

The literature search was performed using the databases MEDLINE via PubMed (from 1966) and EMBASE via Ovid (from 1974) on the 8^th^ of May 2018. Reference lists of the included systematic reviews were screened to capture relevant systematic reviews that were not found during the initial search. The following key words were used: “meat”, “review”, “meta-analysis”. The search was limited to title/abstract, the filter “humans” was used, and only English literature was considered. Moreover, restrictions were made for the following key words to exclude systematic reviews examining meat that has been contaminated with i.e. campylobacter, or other kind of zoonosis or pathogens: “zoonotic”, “pathogen”, “bacteriocins”, “microbial”, “antimicrobial”, “campylobacter”, “contamination”, “contaminated”, “food allergy”. The search strategy is presented in **S1 Table**.

### Study selection

The selected systematic reviews, including systematic reviews of systematic reviews, and meta-analyses were imported to the reference management software, EndNote X7.4, and duplicates were removed. We further only considered reviews that examined a healthy adult population (≥18 years) at baseline, and thus reviews restricted to specific patient populations were excluded. The primary outcomes were incidence and/or mortality of any cancers, T2D, and any CVD; thus reviews concerning other disease outcomes where excluded. There were no specific restrictions regarding the definition of processed meat. If reviews mentioned “processed meat” and/or refereed to processing methods (i.e. salted meat) and/or listed processed meat products (bacon, ham, sausages, luncheon meats, etc.), they were considered eligible for inclusion. Reviews that presented combined results on processed meat with other types of meat, e.g. fresh red meat (unprocessed beef, pork, lamb, etc.), were excluded.

Full-text versions were obtained and examined for any review that appeared to meet the inclusion criteria based on the title/abstract, or where a definite decision could not be made based on the title/abstract alone.

### Quality assessment and data extraction

Full-texts of the selected systematic reviews and meta-analyses were appraised using AMSTAR criteria [19,20]. AMSTAR is an 11-item tool to assess the general methodological quality of systematic reviews [19,20]. This tool has been internally and externally validated and has been found to have good reliability [21]. Based on the tool, a score was calculated were each AMSTAR item met give 1 point and the maximum score is 11. A score from 0–4 indicates low quality, 5–8 moderate quality, and 9–11 high quality, which is the most frequently used categorization method [22].

We excluded systematic reviews and meta-analyses that did not assess or document the scientific quality of the included primary studies (did not meet AMASTAR item number 7) as well as those reviews that did not use at least two electronic sources in their search strategy (did not meet AMSTAR item number 3) [19].

Subsequently, descriptive information of the remaining included systematic reviews was extracted using a predefined structured form developed for this review, separately for the three main outcomes and their risk factors. The information was related to study design, study population, exposure characteristics, number of included studies, authors’ conclusions, and funding. As part of the assessment of the internal validity of the systematic reviews, we further aimed to explore potential reasons for heterogeneous results in meta-analyses by considering variations in factors such as study design and quality. The results were summarized for each cancer type, T2D, and CVD.

*Post hoc* evaluations of the certainty in the estimates of the individual outcomes of interest were rated using Grading of Recommendations, Assessment, Development and Evaluations (GRADE) method [23]. Four possible ratings of the quality were available: high, moderate, low, and very low. Downgrading was done, by investigating the following five domains: 1) risk of bias; 2) inconsistency; 3) indirectness; 4) imprecision and 5) publication bias.

## Results

### Study selection

**Fig 1:**
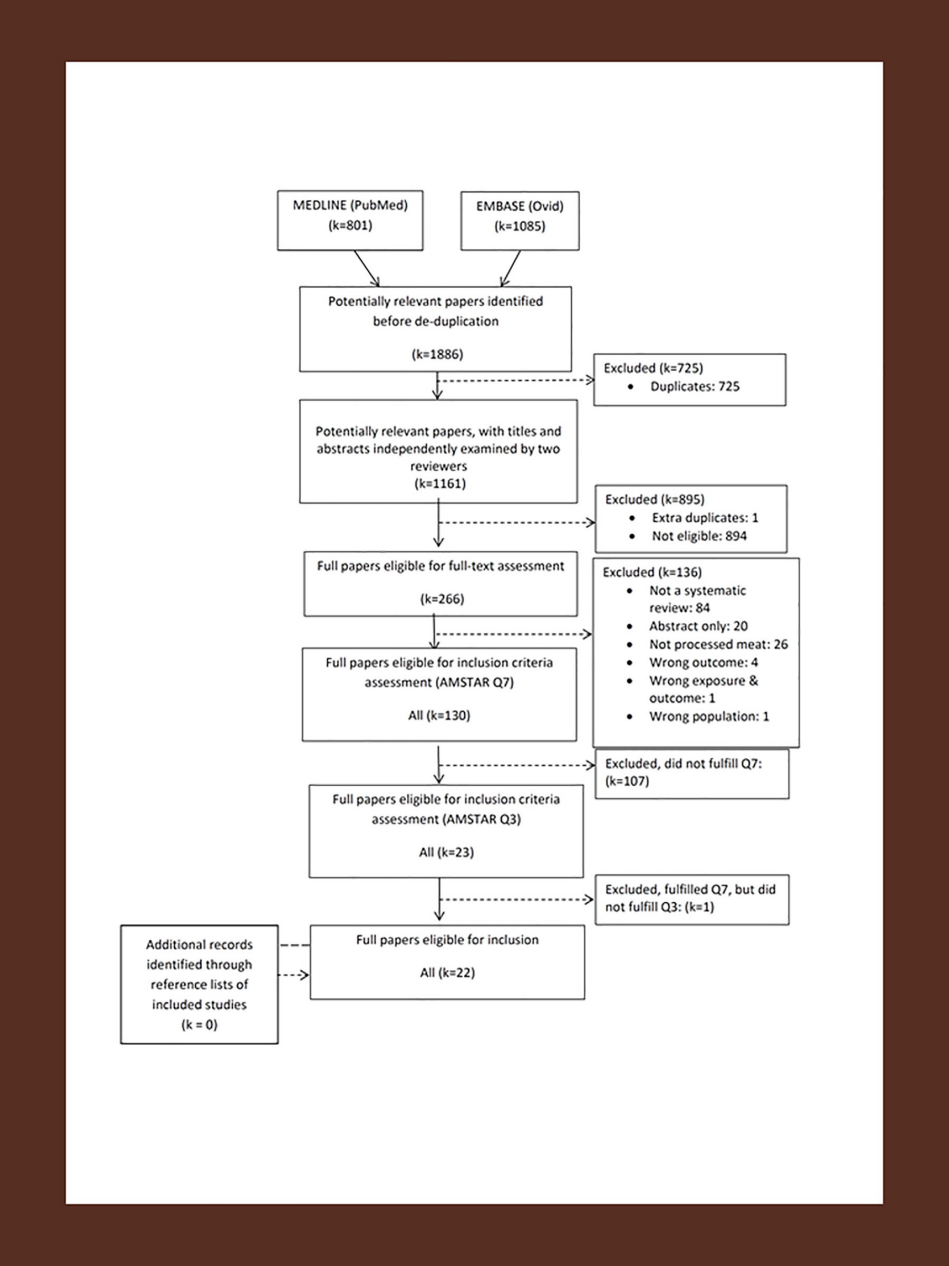
PRISMA flow diagram overview of existing systematic reviews on processed meat and health.

After removing duplicates, the literature search resulted in 1,161 records (flowchart presented in **Fig 1**). Of these, 1 extra duplicate was identified and 894 papers were excluded during the title and abstract screening due to not being relevant for the present review, which resulted in 266 papers extracted for full-text assessment.

During the full-text assessment, we excluded reviews that: were not systematic reviews (k = 84); were conference abstracts (k = 20); did not present results for processed meat alone, i.e. separate the results of processed meat from total meat intake or fresh red meat intake (k = 26); did not include cancer, T2D or CVD as outcomes (k = 4), included the wrong population (individuals with T2D) (k = 1), did not perform a quality assessment of primary studies (AMSTAR Q7, k = 107) and did not perform a comprehensive search (AMSTAR Q3, k = 1), thus resulting in a total of 22 eligible systematic reviews [13,15,24–43]. A list of the reviews excluded during the full-text assessment, which includes the reason for exclusion for each review, can be found in **S2 Table**.

To alleviate any concerns regarding inaccurate conclusions that may be drawn by not including all existing reviews, we also performed a post hoc quality assessment of one additional systematic review that did not perform a comprehensive search but did assess quality.

### Description of included systematic reviews

Out of the 22 eligible systematic reviews, 20 included meta-analyses and 2 were systematic reviews of systematic reviews. The studies were all published between 2010 and 2017. The characteristics of all the studies, sorted by the outcome, are summarized in **Tables 1–5**, and the results of the meta-analyses are presented in **Tables 6 and 7**. An overview of all results from the subgroup analyses are presented in **S5 Table**.

**Table 1:** Summary characteristics of included systematic reviews (SR) on processed meat and cancer.

First author	Databases searched		No. studies (case-control/cohort)	No. participants Cases/controls or cohort size	Study design	Exposure	Subgroup analyses	Authors’ conclusion	Funding and conflict of interest stated^a^	AMSTAR score^b^
**Esophageal cancer**										
Choi (2013)	Pubmed and Embase		18 (15/3)	Case-control: 3851/10064 Cohort: 1162/1137288	SR and meta-analysis	Processed meat	Study design, histological subtype, study location, sex, or study quality, adjustments	(+)	No	6
Zhu (2014)	Medline (PubMed), Embase, Cochrane Library		15 (12/3)	Case-control: 8934/21504 Cohort: 4379/1897574	SR and meta-analysis	Processed meat	Study quality, Study design, control source, geographic region, adjustments	(+)	No	6
**Nasopharyngeal carcinoma**										
Li (2016)	PubMed, Embase, Google Scholar, CNKI (Chinese), Wanfang (Chinese)		13 (13/0)	5849/12735	SR and meta-analysis	Processed meat	Dose	(+)	No	5
**Pancreatic cancer**										
Zhao (2017)	PubMed, Embase, Web of Science		20 (6/14)	Case-control: 1780 (calculated) / 4243 (calculated) Cohort: 8092 (calculated) / 3451 636 (calculated)	SR and meta-analysis	Processed meat	Gender, geographic area, sample size, publication year, quality score, adjustments	Case-control: (+) Cohort: (x)	No	5
**Hepatocellular carcinoma**										
Lou (2014)		PubMed, Web of Science and EMBASE	5 (2/3)	1670093 (All)	SR and meta-analysis	Processed meat	Not conducted due to small sample size	(x)	Yes	5
**Gastric cancer**										
Fang (2015)		Medline, Embase, Web of Science	Processed meat: 7 (0/7) ham-bacon- sausage: 5 (0/5)	Processed meat: 3243/2002100 Ham- bacon- sausage: 1573 /321858	SR and meta-analysis	Processed meat, Ham-bacon- sausage	Dose-response, Geographical location, Anatomical subsites	(+)	No	6
Li (2012)		The Cochrane Library, PubMed, Embase, ISI Web of Knowledge, China Academic Journal Network Publishing Database, and Chinese Scientific Journals Full text Database and Chinese Biomedical Literature Database	2 SR	N/A	SR of SR	Processed meat	None	(+)	Yes	6
Zhao (2017)		PubMed, Embase	33 (25/8)	Case-control: 8286 (calculated) / 57319 (calculated) Cohort: 2148 (calculated) / 1262355 (calculated)	SR and meta-analysis	Processed meat	Subtype of gastric cancer	Case- control: (+) Cohort: (x)	No	6
Zhu (2013)		Medline (PubMed), Embase, Cochrane Library	26 (17/9)	Case-control: 11680/67544 Cohort: 5118/2343450	SR and meta-analysis	Processed meat	Type of processed meat, study quality, study design, sex, histologic subtype, anatomical subtype, geographic region, outcome, adjustments	(+)	No	7
**Glioma**										
Quach (2016)		Pubmed, Medline, Embase, CINAHL, PsychINFO, AARP Ageline, TOXLINE, HuGEnet, Cochrane Library	Cured meat: 1 SR	N/A	SR of SR	Cured meat, hot dogs, bacon, ham	None	(+)	Yes	7
Saneei (2015)		PubMed/Medline, ISI Web of Knowledge, Excerpta Medica database, Ovid database, Google scholar, Scopus	17 (13/4)	Case-control: 4174 (calculated) /10405 (calculated) Cohort: 957 (calculated)/81457 (calculated)	SR and meta-analysis	Processed meat	Study design, Main outcome. Energy adjustment status, Dietary assessment tools, Study quality	(x)	No	8
**Ovarian cancer**										
Wallin (2011)		PubMed, Embase	5 (0/5)	Cohort: 2062 (calculated) / 648931 (calculated)	SR and meta-analysis	Processed meat	Dose	(x)	No	6
**Non-Hodgkin lymphoma**										
Solimini (2016)	PubMed, Scopus		11 (8/3)	Case-control: 4386 (calculated) /12573 (calculated) Cohort: 4982 (calculated) /938439 (calculated)	SR and meta-analysis	Processed meat	Study design, Histological subtype, geographic area, Study adjustment, Stepwise exclusion of studies	(o)	No	7
Yang (2015)		Medline, Embase	20 (13/3)	Case-control: 9060 (calculated) /23941 (calculated) Cohort: 5049 (calculated) /810603 (calculated)	SR and meta-analysis	Processed meat	Study design, Sex, Country, Type of FFQ, study quality, adjustments	(+)	No	7
**Lung cancer**										
Yang (2012)		Medline (PubMed), Embase, Web of Science	10 (NR)	NR	SR and meat-analysis	Processed Meat	Study quality, Study design, Gender	(x)	No	7
**Oral cavity and orophanx cancer**										
Xu (2014)		PubMed, Embase, Cochrane Library Central database	9 (9/0)	4104/501730	SR and meta-analysis	Processed Meat	Continent, Adjustments	(+)	No	7
**Renal cell carcinoma**										
Zhang (2017)		Medline and Embase	19 (15/4)	Case-control: 10668 (calculated) / 26979 (calculated) Cohort: 4033 (calculated) / 1757161 (calculated)	SR and meta-analysis	Processed Meat	Location, study design, FFQ type, available exposure data, study quality score, number of cases, and adjustments	(+)	No	6
**Colorectal cancer**										
Zhao (2017)		Pubmed and Embase	23 (11/12)	Case-control: 8729 (calculated) / 13363 (calculated) Cohort: 15745 (calculated) / 1555178 (calculated)	SR and meta-analysis	Processed Meat	Geographic area, sample size, publication year, quality score, questionnaires used and adjustments	Case-control: (+) Cohort: (+)	No	6
**Cancer mortality**										
O´Sulivan (2013)		Cochrane Library, Medline, Embase, ProQuest, ProQuest dissertations	3 (0/3)	677517	SR and meta-analysis	Processed meat	(Quality assessment), (Ethnicity)^c^	(+)	No	7
Wang (2016)		Medline, Embase, ISI Web of Knowledge, CINAHL, Scopus, Cochrane Library	5 (0/5)	45738/1144264	SR and meta-analysis	Processed meat	Location, Gender, follow-up time, study quality, number of participants	(+)	No	7

^a^ Both within the SR and the included studies

^b^ Maximum AMSTAR score is 11 for meta-analysis and 9 for reviews

^c^ Sensitivity analysis including/excluding studies in relation to quality of studies and ethnicity of study population.

(+) association; (x) no association; (o) no conclusion. Abbreviations: NR: not reported; SR: systematic review

**Table 2:** Summary characteristics of included systematic reviews (SR) on processed meat and diabetes type II.

First author	Databases searched	No. studies (case-control/cohort)	No. participants Cases/controls or cohort size	Study design	Exposure	Subgroup analyses	Authors’ conclusion	Funding and conflict of interest stated^a^	AMSTAR score^b^
Micha (2010)	Medline, Embase, Agris, AMED, HMIC, sycINFO, Cochrane Library, Web of Knowledge, CABI, CINAHL, conference abstracts (ZETOCH)	7 (0/7)	1097/218380	SR and meta-analysis	Processed meat		(+)	No	7

^a^ Both within the SR and the included studies

^b^ Maximum AMSTAR score is 11 for meta-analysis and 9 for reviews.

(+) association; (x) no association; (o) no conclusion. Abbreviations: NR: not reported; SR: systematic review

**Table 3:** Summary characteristics of included systematic reviews (SR) on processed meat and coronary heart disease.

First author	Databases searched	No. studies (case-control/cohort)	No. participants Cases/controls or cohort size	Study design	Exposure	Subgroup analyses	Authors’ conclusion	Funding and conflict of interest stated^a^	AMSTAR score^b^
Micha (2010)	Medline, Embase, Agris, AMED, HMIC, PsycINFO, Cochrane Library, Web of Knowledge, CABI, CINAHL, conference abstracts (ZETOCH)	5 (1/4)	23889/218380	SR and meta-analysis	Processed meat	-	(+)	No	7

^a^ Both within the SR and the included studies

^b^ Maximum AMSTAR score is 11 for meta-analysis and 9 for reviews.

(+) association; (x) no association; (o) no conclusion. Abbreviations: NR: not reported; SR: systematic review

**Table 4:** Summary characteristics of included systematic reviews (SR) on processed meat and stroke.

First author	Databases searched	No. studies (case-control/cohort)	No. participants Cases/controls or cohort size	Study design	Exposure	Subgroup analyses	Authors’ conclusion	Funding and conflict of interest stated^a^	AMSTAR score^b^
Micha (2010)	Medline, Embase, Agris, AMED, HMIC, PsycINFO, Cochrane Library, Web of Knowledge, CABI, CINAHL, conference abstracts (ZETOCH)	2 (0/2)	2280/218380	SR and meta-analysis	Processed meat	-	(x)	No	7
Kim (2017)	PubMed, Embase, Cochrane Library	5 (0/5)	9522/254742	SR and meta-analysis	Processed meat	Number of cases, follow-up duration, sex, stoke subtypes, and adjustments	(+)	No	6

^a^ Both within the SR and the included studies

^b^ Maximum AMSTAR score is 11 for meta-analysis and 9 for reviews.

(+) association; (x) no association; (o) no conclusion. Abbreviations: NR: not reported; SR: systematic review

**Table 5:** Summary characteristics of included systematic reviews (SR) on processed meat and CVD mortality.

First author	Databases searched	No. studies (case-control/cohort)	No. participants Cases/controls or cohort size	Study design	Exposure	Subgroup analyses	Authors’ conclusion	Funding and conflict of interest stated^a^	AMSTAR score^b^
O´Sulivan (2013)	Cochrane Library, Medline, Embase, roQuest, ProQuest dissertations	4 (0/4)	714647	SR and meta-analysis	Processed meat	(Quality assessment), (Ethnicity)^c^	(+)	No	7
Wang (2016)	Medline, Embase, ISI Web of Knowledge, CINAHL, Scopus, Cochrane Library	6 (0/6)	33278/1195947	SR and meta-analysis	Processed meat	Etnicity/location, gender, follow-up time, quality, number of participants	(+)	No	7

^a^ Both within the SR and the included studies

^b^ Maximum AMSTAR score is 11 for meta-analysis and 9 for reviews

^c^ Sensitivity analysis including/excluding studies in relation to quality of studies and ethnicity of study population. (+) association; (x) no association; (o) no conclusion. Abbreviations: NR: not reported; SR: systematic review

**Table 6:** Evidence from existing meta-analysis on the effect of processed meat and cancer.

First author	Outcome	No. participants (studies) contributing data Cases/controls or cohort size	Meta-analysis result RR (95% CI) (fully adjusted)	Heterogeneity	Publication bias	GRADE
Choi (2013)	Esophageal cancer	Case-control: 3851/10064 Cohort: 1162/1137288	Highest versus lowest category: 1.32 (1.08, 1.62)	I^2^ = 58.4%, P < 0.01 (based on Q and I^2^ statistics)	Egger´s test Case-control: NS Cohort: NS	VERY LOW Due to study design and risk of bias
Zhu (2014)	Esophageal cancer	Case-control: 8934/21504 Cohort: 4379/1897574	Highest versus lowest category: 1.33 (1.04, 1.69)	I^2^ = 61.5%, P < 0.001 (based on Q and I^2^ statistics)	Egger´s test: NS Begg´s test: NS	VERY LOW Due to study design and risk of bias
Li (2016)	Nasopharyngeal carcinoma	5849/12735	low-rank intake of processed meat (<30 g/week): 1.46 (1.31, 1.64)	I^2^ = 61%, P = 0.004 (based on Q and I^2^ statistics)	Egger´s test: NS Begg´s test: NS	VERY LOW Due to study design and risk of bias
Zhao (2017)	Pancreatic cancer	Case-control: 1780 (calculated) / 4243 (calculated) Cohort: 8092 (calculated) / 3451636 (calculated)	Highest versus lowest category: Case-control: 1.62 (1.17, 2.26) Cohort: 1.09 (0.96, 1.23)	Case-control: I^2^ = 58%, P = 0.04 (based on Q and I^2^ statistics) Cohort: I^2^ = 51%, P = 0.001 (based on Q and I^2^ statistics)	Egger´s test: NS Begg´s test: NS	VERY LOW Due to study design, risk of bias and imprecision
Luo (2014)	Hepatocellular carcinoma	1670093 (all)	Highest versus lowest category: 1.01 (0.79, 1.28)	I^2^ = 42.9%, P = 0.136 (based on Q and I^2^ statistics)	Egger´s test: P = 0.07 Begg´s test: NS	VERY LOW Due to study design, risk of bias and imprecision
Fang (2015)	Gastric cancer	3243/2002100	Highest versus lowest category of processed meat consumption: 1.15 (1.03, 1.29)	I^2^ = 8.2%, p-value not reported (based on I^2^ statistics)	Egger´s test: NS Begg´s test: NS	VERY LOW Due to study design, and risk of bias
Fang (2015)	Gastric cancer	1573/321858	Highest versus lowest category of ham, bacon, sausage consumption: 1.21 (1.01,1.46)	I^2^ = 30.6%, p-value not reported (based on I^2^ statistics)	Egger´s test: NS Begg´s test: NS	VERY LOW Due to study design, and risk of bias
Zhao (2017)	Gastric cancer	Case-control: 8286 (calculated) / 57319 (calculated) Cohort: 2148 (calculated) / 1262355 (calculated)	Highest versus lowest categories: Case-control: 1.76 (1.51, 2.05) Cohort: 1.23 (0.98, 1.55)	Case-control: I^2^ = 59%, P = 0.0001 (based on I^2^ statistics) Cohort: I^2^ = 43%, P = 0.09 (based on Q and I^2^ statistics)	Egger´s test: NS Begg´s test: NS	VERY LOW Due to study design, risk of bias and imprecision
Zhu (2013)	Gastric cancer	Case-control: 11680 /67544 Cohort: 5118 /2343450	Highest versus lowest model: 1.44 (1.26, 1.65)*	I^2^ = 61.0%, p<0.001 (based on Q and I^2^ statistics)	Egger´s test: P = 0.04 Begg´s test: NS	VERY LOW Due to study design, and risk of bias
Saneei (2015)	Glioma	Case-control: 4174 (calculated) /10405 (calculated) Cohort: 957 (calculated)/ 810457 (calculated)	Highest versus lowest category: 1.14 (0.98, 1.33)	I^2^ = 50.6%, P = 0.006 (based on Q and I^2^ statistics)	Egger´s test: P = 0.07 Begg´s test: NS	VERY LOW Due to study design, risk of bias and imprecision
Wallin (2011)	Ovarian cancer	Cohort: 2062 (calculated)/648931 (calculated)	Increment in 100 g/week: 1.05 (0.98, 1.14)	I^2^ = 0.0%, P = 0.65 (based on Q and I^2^ statistics)	Egger´s test: NS	VERY LOW Due to study design, risk of bias and imprecision
Solimini (2016)	Non-Hodgkin lymphoma	Case-control: 4386 (calculated)/12573 (calculated) Cohort: 4982 (calculated)/ 938439 (calculated)	Highest versus lowest intake: 1.06 (0.98, 1.15)	I^2^ = 3.6%, P = 0.41 (based on Q and I^2^ statistics)	Egger´s test: NS	VERY LOW Due to study design, risk of bias and imprecision
Yang (2015)	Non-Hodgkin lymphoma	Case-control: 9060 (calculated)/23941 (calculated) Cohort: 5049 (calculated)/810603 (calculated)	Highest versus lowest intake: 1.17 (1.07, 1.29)	I^2^ = 37.1%, P = 0.057 (based on Q and I^2^ statistics)	Egger´s test: NS	VERY LOW Due to study design, and risk of bias
Yang (2012)	Lung cancer	Not reported	Highest versus lowest category: 1.06 (0.90, 1.25)	I^2^ = 79.5%, P<0.001 (based on I^2^ statistics)	Egger´s test: NS Begg´s test: NS	VERY LOW Due to study design, risk of bias and imprecision
Xu (2014)	Oral cavity and orophanx cancer	4104/501730	Highest versus lowest category: 1.91 (1.19, 3.06)	I^2^ = 85.9%, P<0.001 (based on Q and I^2^ statistics)	Egger´s test: NS Begg´s test: NS	VERY LOW Due to study design, and risk of bias
Zhang (2017)	Renal cell carcinoma	Case-control: 10668 (calculated) / 26979 (calculated) Cohort: 4033 (calculated) / 1757161 (calculated)	Highest versus lowest level: 1.13 (1.03–1.24)	I^2^ = 45.6%, P = 0.014 (based on I^2^ statistics)	Egger´s test: NS Begg´s test: NS	VERY LOW Due to study design, and risk of bias
Zhao (2017)	Colorectal cancer	Case-control: 8729 (calculated) / 13363 (calculated) Cohort: 15745 (calculated) / 1555178 (calculated)	Highest versus lowest categories: Case-control: 1.36 (1.09, 1.69) Cohort: 1.15 (1.07, 1.24)	Case-control: I^2^ = 76%, P<0.00001 (based on Q and I^2^ statistics) Cohort: I^2^ = 27%, P = 0.18 (based on Q and I^2^ statistics)	Egger´s test: NS Begg´s test: NS	VERY LOW Due to study design, and risk of bias
O´Sulivan (2013)	Cancer mortality	677517	1.13 (1.09, 1.17)	I^2^ = 0%, P = 0.99 (based on I^2^ statistics)	Test: not reported P-value: not reported Visual inspection: indication of publication bias	VERY LOW Due to study design, and risk of bias
Wang (2016)	Cancer mortality	45738/1144264	Highest versus lowest consumption: 1.08 (1.06, 1.11)	I^2^ = 0%, P = 0.450 (based on Q and I^2^ statistics)	Egger´s test: NS Begg´s test: NS	VERY LOW Due to study design, and risk of bias

* RR reported in the meta-analysis is different from the one reported in the text; NS: Non-significant

**Table 7:** Evidence from existing meta-analysis on the effect of processed meat and diabetes type II, CHD, stroke and CVD mortality.

First author	Outcome	No. participants (studies) contributing data Cases/controls or cohort size	Meta-analysis result RR (95% CI) (fully adjusted)	Heterogeneity	Publication bias	GRADE
Micha (2010)	Diabetes Mellitus	1097/218380	Per 50g/day of processed meat: 1.19 (1.11, 1.27)	I^2^ not reported; P<0.001 (based on X^2^ statistics)	Not reported	VERY LOW Due to study design, and risk of bias
Micha (2010)	Coronary heart disease	23889/218380	Per 50g/day of processed meat: 1.42 (1.07, 1.89)	I^2^ not reported; P = 0.04 (based on X^2^ statistics)	Begg´s test: NS	VERY LOW Due to study design, and risk of bias
Micha (2010)	Stroke	2280/218380	Per 50g/day of processed meat: 1.14 (0.94, 1.39)	Not reported	Not reported	VERY LOW Due to study design, risk of bias and imprecision
Kim (2017)	Stroke	9522/254742	Highest versus lowest category: 1.17 (1.08, 1.25)	I^2^ = 0.0%, P = 0.510 (based on I^2^ statistics)	Egger´s test: NS	VERY LOW Due to study design, and risk of bias
O´Sulivan (2015)	CVD-related mortality	714647	1.17 (1.02, 1.33)	I^2^ = 88%, P<0.001 (based on I^2^ statistics)	Not reported	VERY LOW Due to study design, and risk of bias
Wang (2016)	CVD-related mortality	33278/1195947	Highest versus lowest consumption: 1.15 (1.07, 1.24)	I^2^ = 75.4%, P<0.001 (based on Q and I^2^ statistics)	Egger´s test: NS Begg´s test: NS	VERY LOW Due to study design, and risk of bias

* RR reported in the meta-analysis is different from the one reported in the text; NS: Non-significant

### Quality assessment of included systematic reviews

None of the 22 included systematic reviews received the maximum AMSTAR score (**Table 1–6**; detailed quality scores provided in **S3 Table**). Based on the AMSTAR criteria they were all classified to be of moderate quality, with a mean value of 6.36 and a range from 5 to 8. The items within the AMSTAR scoring systems that were most infrequently fulfilled across the included reviews were the AMSTAR item number 1,4, 8 and 11. The AMSTAR item number 1 concerned whether an “a priori” design was provided (i.e. referred to development of a protocol, ethics approval, or a predetermined/a priori published research objectives), and only one out of the 22 reviews fulfilled that [35]. The AMSTAR item number 4 referred whether the status of publication (grey literature / unpublished literature) was used as an inclusion criterion. To receive a “yes” in this item, the authors should state that they searched for reports regardless of their publication type, in other words that they searched for “grey literature” or “unpublished literature”. There were only 3 out of 22 systematic reviews that fulfilled this item [15,30,34]. The AMSTAR item number 8 referred to whether the scientific quality grading was used appropriately in formulating conclusions, i.e. describing the quality of the evidence [35], and only 5 out of 22 systematic reviews fulfilled that [30,33,34,39,40]. All reviews assessed publication bias, when applicable, and overall there were no indication of such bias, regardless of test, except in three reviews (on hepatocellular carcinoma [32], gastric cancer [41] and cancer mortality [33] that all favored significant results (**Tables 6 and 7**). The AMSTAR item number 11 considered whether the potential conflict of interest was addressed. To receive a “yes” for this item, conflict of interest needs to be addressed both for each of the included studies, and for the systematic review itself. There were only two out of 22 systematic reviews that addressed conflict of interest appropriately according to AMSTAR [30,34]. A narrative AMSTAR evaluation per outcome has provided in **S6 Table**.

In accordance to the GRADE approach, results from observational studies (case-control and cohort studies) are by default considered to be of low quality, yet the quality may be upgraded to moderate if there are no issues with confounding, there is a large effect, and/or there is a consistent dose-response relationship. Since this was not the case, as residual confounding is always suspected to be present in observational studies, and the effect and the dose-response pattern were modest or not significant, the certainty of the effect estimate was downgraded to very low, due to serious risk of bias and/or serious imprecision. There were issues with inconsistency (heterogeneity) in more than half of the included meta-analyses (reported in **Table 4**), but there were no issues regarding indirectness or publication bias (**Tables 6 and 7**).

### Qualitative synthesis of included systematic reviews on cancers

Most of the included systematic reviews concerned different types of cancer (19 out of 22 systematic reviews).

#### Esophageal cancer

Both reviews indicated an overall association between processed meat intake and risk of esophageal cancer with a summary relative risk (RR) estimate for the highest versus the lowest categories of processed meat intake of 1.32 (95% CI: 1.08, 1.62) and 1.33 (95% CI: 1.04, 1.69) in the meta-analyses by Choi et al. [28] and Zhu et al. [42], respectively (**Table 6**)). Choi et al. based their meta-analysis on 15 case-control studies and 3 cohort studies [28], whereas Zhu et al. included 12 case-control studies and 3 cohort studies [42]. Both meta-analyses showed a large degree of heterogeneity of approximately 60% (**Table 6**). Nine of the case-control studies, and the 3 cohort studies were overlapping in the meta-analyses of the systematic reviews (**S4 Table**). In Choi et all [28], they reported meta-analyses subdivided by study design showing that the direct associations between a high processed meat intake and risk of esophageal cancer remained for the case-control studies (RR: 1.36, 95% CI: 1.07, 1.74) but not for the cohort studies (RR: 1.25, 95% CI: 0.83, 1.86).

#### Head and neck cancer (nasopharyngeal carcinoma)

The results of this review [31] were based on 13 case-control studies, and suggested an increased risk of nasopharyngeal carcinoma among individuals with low intake of processed meat (<30 g/week) compared to those never eating processed meat (RR: 1.46; 95% CI: 1.31, 1.64; **Table 6**). The meta-analysis also showed heterogeneity across the primary studies (I^2^ = 61%, p = 0.004; **Table 6**).

#### Pancreatic cancer

The review included 6 case-control studies and 14 cohort studies [27] and reported that processed meat consumption (highest versus lowest category) was positively associated with pancreatic risk in case-control studies (RR: 1.62; 95% CI: 1.17, 2.26) [27], however no association was observed in cohort studies (RR: 1.09; 95% CI: 0.96, 1.23) [27] (**Table 6**). The meta-analysis showed substantial heterogeneity among both the case-cohort studies (I^2^ = 58%) and the cohort studies (I^2^ = 51%; **Table 6**).

#### Liver cancer (hepatocellular carcinoma)

Processed meat intake was not associated with liver cancer in this meta-analysis (RR 1.01; 95% CI: 0.79, 1.28; **Table 6**), based on 2 case-control studies and 3 cohort studies. There was no significant heterogeneity across the studies (**Table 6**).

#### Gastric cancer

Three of the studies [29,30,41] concluded that there was an association between processed meat intake and gastric cancer risk; while one study (Zhao et al.) concluded the same in case-control studies, but found no association in cohort studies [26] (**Tables 1 and 6**). The meta-analysis by Fang et al. included only cohort studies (k = 12) and found a RR of 1.15 (95% CI: 1.03, 1.29; neither I^2^ nor a p-value for the test of homogeneity was not reported) [29], whereas the meta-analysis by Zhu et al. included 17 case-control studies and 9 cohort studies (**S4 Table**), and found a RR 1.45 (1.26, 1.65), with high degree of heterogeneity across the studies (I^2^ = 61.0%) [41]. In addition, Zhao et al. included in their meta-analysis 25 case-control studies and 8 cohort studies and found a RR of 1.76 (95% CI: 1.51, 2.05) with heterogeneity across the studies (I^2^ = 59%) and RR of 1.23 (95% CI: 0.98, 1.55) with moderate heterogeneity (I^2^ = 43%), respectively. Among the cohort studies there were 8 studies overlapping between the Fang et al. and the Zhu et al. reviews and 5 studies overlapping between Fang et al., Zhu et al. and Zhao et al.; whereas there were 6 case-control studies overlapping between Zhu et al. and Zhao et al. reviews (**S4 Table**). In the subgroup analysis, Zhu et al. found similar associations in both the case-control and the cohort studies (**S5 Table**) [41].

#### Brain cancer (glioma)

Based on 13 case-control studies and 4 cohort studies, the meta-analysis in the systematic review by Saneii and colleagues found no association between processed meat intake and glioma risk (RR: 1.14; 95% CI: 0.98, 1.33) [35]. There was some degree of heterogeneity across studies (I^2^ = 50.6%). The systematic review of systematic reviews by Quach and colleagues [34] included one study [44], which found an increased risk of adult glioma in relation to a high cured meat intake.

#### Ovarian cancer

On the basis of 5 cohort studies, the meta-analysis did not suggest an association between processed meat consumption and ovarian cancer (**Tables 1 and 6**)–either by low vs. high intake or in the dose response analyses (**S5 Table**). There was no significant heterogeneity across the primary studies (**Table 6**).

#### Non-Hodgkin lymphoma

The two systematic reviews reached different conclusions: the meta-analysis by Solimini et al. found no association [36], while the meta-analysis by Yang et al. suggested a direct association with risk of Non-Hodgkin lymphoma (RR: 1.17; 95% CI: 1.07, 1.29 for high vs. low processed meat intake) [40]. The meta-analyses from the two reviews included the same 3 cohort studies but the case-control studies differed; out of a total of 14 case-control studies, Solimini et al. included 8 case-control studies and Yang et al. included 13 case-control studies; only 7 case-control studies overlapped between the two reviews. None of the meta-analyses showed significant heterogeneity across the primary studies (**Table 6**). Both meta-analyses found associations in the case-control studies, but not in the cohort studies (**S5 Table**).

#### Lung cancer

In relation to processed meat consumption and lung cancer risk, the included systematic review suggested no association [39]. There was a high degree of heterogeneity across the studies in the meta-analysis (I^2^ = 79.5%).

#### Oral cavity and orophanx cancer

The meta-analysis indicated an association (RR: 1.91; 95% CI: 1.19, 3.06) [38]. Moreover, there was a high degree of heterogeneity across the studies in the meta-analysis (I^2^ = 85.9%; **Table 6**).

#### Renal cell carcinoma

The meta-analysis of this systematic review was based on 15 case-control studies and 4 cohort studies, and found an overall RR of 1.13 (95% CI: 1.03, 1.24) with moderate heterogeneity (I^2^ = 45.6%; **Table 6**) [24].

#### Colorectal cancer

The authors concluded that there was a positive association between processed meat consumption and risk of CRC, which was based on a meta-analysis of 11 case-control studies that found a RR of 1.36 (95% CI: 1.09, 1.69) with considerable heterogeneity (I^2^ = 76%) and on a meta-analysis of 12 cohort studies that found a RR of 1.15 (95% CI: 1.07, 1.24) with low heterogeneity (I^2^ = 27%; **Table 6**).

#### Cancer mortality

The results from O´Sulivan et al. were based on 3 cohort studies [33], and Wang et al. supplemented their systematic review with additional 2 cohort studies [13]; both reviews showed an association between processed meat intake and cancer mortality (O´Sulivan et al.: RR: 1.13, 95% CI: 1.09, 1.17; Wang et al.: RR: 1.08, 95% CI: 1.06, 1.11). Moreover, neither of the meta-analyses showed significant heterogeneity in the meta-analyses (**Table 6**).

### Qualitative synthesis of included systematic reviews on diabetes

The result of the meta-analysis suggested an association (RR: 1.19; 95% CI: 1.11, 1.27), with significant heterogeneity (p<0.001; **Table 7**). The meta-analysis was conducted based on 7 cohort studies.

### Qualitative synthesis of included systematic reviews on coronary heart disease

According to the results of the meta-analyses, increased processed meat intake was associated with risk of CHD (RR: 1.42; 95% CI: 1.07, 1.89 (heterogeneity: p = 0.04)) [15]. The results were based on 1 case-control study and 4 cohort studies (**Table 3**).

### Qualitative synthesis of included systematic reviews on stroke

In regard to stroke, Kim et al. reported an association (RR of 1.17 (95% CI: 1.08, 1.25) with low heterogeneity (I^2^ = 0.0%) [43], however Micha et al. did not (RR of 1.14 (95% CI: 0.94, 1.39; heterogeneity not analyzed) [15] (**Table 4**). The results were based on 2 cohort studies for Micha et al. [15] and 5 cohort studies for Kim et al. [43], with none of the studies overlapping (**S4 Table**).

#### Results from post hoc quality assessment analyses

Its meta-analysis [45] was based on 3 cohort studies, with only one overlapping with the study of Micha et al. [15] and found that processed meat consumption increased the risk of stroke (RR: 1.17 1.09, 1.27)) [45].

### Qualitative synthesis of included systematic reviews on CVD mortality

The results on CVD mortality (O´Sulivan et al. [33] showed an association: RR: 1.17; 95% CI: 1.02, 1.33); Wang et al. [13]: RR: 1.15; 95% CI: 1.07, 1.24). WhileThe total number of cohort studies included in the meta-analysis of O´Sulivan et al. [33] and Wang et al. [13] was 7, but only three studies overlapped (**S4 Table**), and the meta-analyses showed a large degree of heterogeneity (**Table 7)**.

## Discussion

In this overview, we assessed the quality alongside summarizing the results of published systematic reviews and meta-analyses that examined associations between processed meat consumption and the risk of multiple chronic diseases. We assessed the methodological quality of published systematic reviews and meta-analyses using the AMSTAR tool, and found a suboptimal quality of most previous reviews. Associations were more often found when reviews were based on results from case-control than when based on cohort studies, suggesting that the better the study design, the lower the probability of an association.

According to GRADE, the quality of evidence was very low for the individual outcomes. The rating was based on observational study design, in combination with serious risk of bias, and/or serious imprecision.

In the study selection process, we excluded 107 reviews that did not assess and document the scientific quality of the included studies (AMSTAR item number 7). In line with other authors [16,17], we did this to ensure that the included reviews were ‘systematic’. The AMSTAR score of these reviews were already short of two points, but to accommodate any concerns regarding inaccurate conclusions that may be drawn by not including all published reviews, we performed a post hoc quality assessment of the additional systematic review that was “a priori” not included because only one database was searched, but did assess quality of the primary studies (AMSTAR item number 3). As anticipated, the AMSTAR score for this single review was moderate. Whether searching one database captures most of the existing literature is debatable. Coverage by the most commonly used databases has earlier been shown to be high (>90%). However, recall estimates (defined as the percentage of relevant records retrieved divided by the total number of included studies in the individual systematic reviews), even for the best performing databases (EMBASE/MEDLINE), have been shown to be insufficient in retrieving references for systematic reviews (< 50%), when the databases were used alone [46]. In the following, we discuss in more detail the main results from the included reviews by disease outcomes, considering the designs of the primary studies as a quality indicator.

### Cancers

The results of the included systematic reviews and meta-analyses suggested that overall a high intake of processed meat was related to an increased risk of esophageal cancer, nasopharyngeal carcinoma, gastric cancer, oral cavity, renal cell carcinoma, CRC, and cancer mortality. Except for gastric cancer and total cancer mortality, the evidence for an association between processed meat intake and the cancers, seemed to a large degree to be driven by results from case-control studies rather than cohort studies. The same was true for results on pancreatic cancer. As pointed out in several earlier studies, interpretation of results based on case-control studies only should be done cautiously, because of their well-known methodological limitations.

Cancer in the liver, brain (glioma), ovaries, lung and PCC did not seem to be associated with intake of processed meat. For the most part, these results were consistent across studies with different designs. Regarding Non-Hodgkin lymphoma, there were discrepancies in the results from the two included meta-analyses, which may be explained by the fact that the primary studies differed between the two meta-analyses. Also, the increased risk of Non-Hodgkin lymphoma with high processed meat intake seemed to be driven by the results from the case-control studies. The two meta-analyses that examined processed meat intake and risk of Non-Hodgkin lymphoma included the same cohort studies. Considering shortcomings of case-control studies, the conclusion based on the meta-analysis by Solimini et al. [36] suggesting no association between processed meat intake and Hodgkin lymphoma may be most valid.

### Diabetes and cardiovascular disease

An association was observed between a high intake of processed meat and risk of T2D, along with CVD incidence and mortality. For processed meat intake and risk of stroke, one study did not find an association [15], while the other did [43]. Most of the primary studies on processed meat intake and risk of diabetes and cardiovascular diseases that were in the meta-analyses were based on cohort studies, albeit limited to few (diabetes: k = 7; CHD: k = 5; stroke: k = 2 and k = 5; CVD mortality: k = 4 and k = 6).

### Strengths and limitations

A strength of the present overview lays in the systematic quality assessment of multiple reviews examining the influence of processed meat on three main common chronic diseases. However, there are inherited limitations when conducting overviews, umbrella reviews or ‘reviews of reviews’. The conclusion on the outcomes here relies on the methodological quality of primary study selection, specific eligibility criteria, and adequacy of the reporting in systematic reviews. Thus, even well-conducted systematic reviews may have relied on an evidence base that was poor or limited from the primary studies included. Moreover, relevant important results from primary studies may be lacking in this present synthesis, due to the stringent study selection criteria of the included systematic reviews, or because important primary studies may have been published after the search date in the systematic reviews [47].

Following the guidelines of the AMSTAR quality assessment tool in the present study, we registered our protocol prior to the search; further, the study selection and quality assessment were performed independently by two researchers. We performed a comprehensive literature search, however we only selected published systematic reviews. Since published reviews are systemically different from unpublished reviews, issues regarding publication bias should also be kept in mind, especially because of the inability to capture these quantitatively for systematic reviews.

#### Limitations of the quality assessment tool AMSTAR

A number of limitations of the AMSTAR tool have previously been emphasized [20,48]. First, the AMSTAR version we used did not specifically address systematic reviews of non-randomized studies. Such a tool (AMSTAR 2, which was recently released) was being developed at the time this work was conducted. Secondly, the AMSTAR relies on what information is available and reported in the systematic review, which makes evaluation of especially older systematic reviews prone to a low score, i.e. the methodological quality may be underestimated. We did not contact the review authors in attempt to avoid false-negative results. Other tools, such as ROBIS, may be considered more applicable for this purpose [49], although many of the signaling questions between AMSTAR, AMASTAR 2 and ROBIS overlap. The reasons for selecting AMSTAR were that AMSTAR is valid, reliable, easy to use, with high inter-rater agreement and a widely used instrument [21], also for research on diet and health [50–52]. Indeed, previous studies show that inter-rater agreement in AMSTAR is higher compared to ROBIS (AMSTAR > 80% versus ROBIS ≈ 60%) [53]. Even though a formal comparison of results between the two tools of risk of bias may be of interest for future research; systematic reviews in other research fields have already shown good consistencies between AMSTAR and ROBIS, i.e. reviews that showed low quality on AMSTAR also tended to demonstrate high risk of bias on ROBIS [53,54].

## Conclusions

Many previous reviews report adverse associations between a high processed meat intake and risk of various cancers, T2D and CVD, but most were of moderate methodological quality, where evidence for associations were more often found when reviews were based on results from case-control than when based on cohort studies, suggesting that the better the study design, the lower the probability of an association. Moreover, the overall certainty in the evidence was very low across all individual outcomes, due to serious risk of bias and imprecision.

A systematic quality assessment of each of the primary studies in a review should be performed in future systematic reviews prior to formulating a concrete conclusion of the evidence.

## Supporting information

S1 Table Search strategy in MEDLINE via PubMed and EMBASE via Ovid. (DOCX)

S2 Table Excluded studies after full-text assessment. (DOCX)

S3 Table Quality assessment using AMSTAR. (DOCX)

S4 Table Study matrix. Matrix showing the studies (left column) that the estimates (top row) are based on.
(DOCX)

S5 Table Subgroup analyses relevant to this SR. (DOCX)

S6 Table A narrative AMSTAR evaluation per outcome. (DOCX)

S7 Table PRISMA 2009 checklist. (DOC)

## Abbreviations

AMSTAR: Assessing the Methodological Quality of Systematic Reviews
CVD: Cardiovascular disease
CI: confidence interval
GRADE: Grading of Recommendations Assessment, Development and Evaluation
IARC: International Agency for Research on Cancer
PROSPERO: International Prospective Register of Systematic Reviews
NOS: Newcastle-Ottawa Scale
PRISMA: Preferred Reporting Items for Systematic Reviews and Meta-Analyses
RR: relative risk
T2D: type II diabetes
WHO: World Health Organization
