# Supplementary material for: Processed meat intake and chronic disease morbidity and mortality: An overview of systematic reviews and meta-analyses
Source: PLoS One. 2019 Oct 17;14(10):e0223883. doi: 10.1371/journal.pone.0223883 (PMC6797176; doi:10.1371/journal.pone.0223883)
Supplement: S1 Table — (DOCX) [file pone.0223883.s001.docx]

**Supplemental table 1- Search strategy in MEDLINE via PubMed and EMBASE via Ovid**

| Database | **Search term** | **Comments** | | |
| --- | --- | --- | --- | --- |
|  |  | **Filters / Limits** | **Date / Time of the search** | **Total hits** |
| MEDLINE via PubMed | (meat[Title/Abstract]) NOT (zoonotic[Title/Abstract] OR pathogen?[Title/Abstract] OR bacteriocins[Title/Abstract] OR microbial[Title/Abstract] OR antimicrobial[Title/Abstract] OR campylobacter[Title/Abstract] OR contamination[Title/Abstract] OR contaminated[Title/Abstract] OR “food allergy” [Title/Abstract]) AND (review[Title/Abstract] OR meta-analysis[Title/Abstract]) | human + English | 2018.05.08 / 16:02 | 801 |
|  |  |  |  |  |
| EMBASE via Ovid | ((meat not (zoonotic or pathogen? or bacteriocins or microbial or antimicrobial or campylobacter or contamination or contaminated or food allergy)) and (review or meta-analysis)).ab,ti. | human + English | 2018.05.08 / 16:05 | 1085 |
|  |  |  |  |  |
