# Supplementary material for: Processed meat intake and chronic disease morbidity and mortality: An overview of systematic reviews and meta-analyses
Source: PLoS One. 2019 Oct 17;14(10):e0223883. doi: 10.1371/journal.pone.0223883 (PMC6797176; doi:10.1371/journal.pone.0223883)
Supplement: S2 Table — (DOCX) [file pone.0223883.s002.docx]

**Supplemental Table 2**. Excluded Studies After Full-Text Assessment.

| **ID** | **Authors** | **Date** | **Title** | **Reasons for exclusion** |
| --- | --- | --- | --- | --- |
| 1 | Abete, I., et al. | 2014 | Association between total, processed, red and white meat consumption and all-cause, CVD and IHD mortality: A meta-analysis of cohort studies | No quality assessment. |
| 2 | Abid, Z., et al. | 2014 | Meat, dairy, and cancer | Not a systematic review. |
| 3 | Ahmed, F. E. | 2004 | Effect of diet, life style, and other environmental /chemopreventive factors on colorectal cancer development, and assessment of the risks | Not a systematic review. |
| 4 | Al Baho, A. K., et al | 2003 | The causative and preventive aspects of diet in cancer | No quality assessment. |
| 5 | Alaejos, M. S., et al. | 2008 | Exposure to heterocyclic aromatic amines from the consumption of cooked red meat and its effect on human cancer risk: a review. | Not a systematic review. |
| 6 | Alexander, D. D., et al. | 2014 | Red meat and colorectal cancer: A quantitative update on the state of the science | Conference abstract (full text is Alexander, 2015). |
| 7 | Alexander, D. D. and C. A. Cushing | 2009 | Quantitative assessment of red meat or processed meat consumption and kidney cancer | No quality assessment. |
| 8 | Alexander, D. D. and C. A. Cushing | 2011 | Red meat and colorectal cancer: a critical summary of prospective epidemiologic studies | No quality assessment. |
| 9 | Alexander, D. D., et al. | 2011 | Meta-analysis of prospective epidemiologic studies of red meat intake and colorectal cancer. | Conference abstract (no article available). |
| 10 | Alexander, D. D., et al. | 2010 | Meta-analysis of prospective studies of red meat intake and colorectal cancer. | Conference abstract (no article available). |
| 11 | Alexander, D. D., et al | 2010 | Processed meat and colorectal cancer: a quantitative review of prospective epidemiologic studies | No quality assessment. |
| 12 | Alexander, D. D., et al | 2010 | A review and meta-analysis of prospective studies of red and processed meat intake and prostate cancer | No quality assessment.    There are sensitivity analyses in regard to selected methodological quality factors, but the “quality result” is not reported for each study. |
| 13 | Alexander, D. D., et al. | 2010 | A review and meta-analysis of red and processed meat consumption and breast cancer | No quality assessment. |
| 14 | Alexander, D. D., et al. | 2011 | Red meat consumption and colorectal cancer: A meta-analysis of prospective epidemiologic studies | Only abstract available, from a congress of epidemiology. |
| 15 | Alexander, D. D., et al. | 2011 | Meta-analysis of prospective studies of red meat consumption and colorectal cancer | No quality assessment. |
| 16 | Alexander, D. D., et al | 2015 | Red Meat and Colorectal Cancer: A Quantitative Update on the State of the Epidemiologic Science | No quality assessment. |
| 17 | Alzalabani, A., et al. | 2015 | Modifiable risk factors of bladder cancer: A quantitative review of meta-analyses | Only abstract available to the European congress of epidemiology. |
| 18 | Al-Zalabani, A. H., et al. | 2016 | Modifiable risk factors for the prevention of bladder cancer: a systematic review of meta-analyses | No quality assessment. |
| 19 | Ames, B. N. and L. S. Gold | 1997 | The causes and prevention of cancer: Gaining perspective | Not a systematic review. |
| 20 | Anand, P., et al | 2008 | Cancer is a preventable disease that requires major lifestyle changes. | Not processed meat. |
| 21 | Andersen, J. J., et al. | 2018 | Red and processed meat consumption and breast cancer: UK Biobank cohort study and meta-analysis | No quality assessment. |
| 22 | Anonymous | 2017 | Exam 1: Association Between Consumption of Red and Processed Meat and Pancreatic Cancer Risk: A Systematic Review and Meta-analysis | Wrong study design. |
| 23 | Aune, D., et al. | 2009 | Meat consumption and the risk of type 2 diabetes: A systematic review and meta-analysis of cohort studies | No quality assessment. |
| 24 | Aune, D., et al. | 2013 | Red and processed meat intake and risk of colorectal adenomas: a systematic review and meta-analysis of epidemiological | No quality assessment. |
| 25 | Aykan, N. F | 2015 | Red meat and colorectal cancer | No quality assessment. |
| 26 | Azeem, S., et al. | 2015 | Diet and Colorectal Cancer Risk in Asia – a Systematic Review | No quality assessment. |
| 27 | Babaei, M., et al | 2010 | Gastric Cancer in Ardabil, Iran - a Review and Update on  Cancer Registry Data | No quality assessment. |
| 28 | Babio, N., et al. | 2009 | Mediterranean diet and metabolic syndrome: the evidence | Not a systematic review. |
| 29 | Baena, R. and P. Salinas | 2015 | Diet and colorectal cancer | No quality assessment. |
| 30 | Baena Ruiz, R. and P. Salinas Hernandez | 2014 | Diet and cancer: Risk factors and epidemiological evidence | No quality assessment. |
| 31 | Bailie, L., et al. | 2017 | Lifestyle Risk Factors for Serrated Colorectal Polyps: A Systematic Review and Meta-analysis. | Not processed meat. |
| 32 | Ballon-Landa, E. and Parsons, J. K. | 2018 | Nutrition, physical activity, and lifestyle factors in prostate cancer prevention | Not a systematic review. |
| 33 | Bandera, E. V., et al | 2007 | Consumption of animal foods and endometrial cancer risk: A systematic literature review and meta-analysis | No quality assessment. |
| 34 | Baxter, A. J., et al. | 2006 | Dietary patterns and metabolic syndrome - A review of epidemiologic evidence. | No quality assessment. |
| 35 | Bella, F., et al. | 2017 | Differences in the association between empirically derived dietary patterns and cancer: a meta-analysis | Not processed meat. |
| 36 | Bener, A. | 2011 | Colon cancer in rapidly developing countries: Review of the lifestyle, dietary, consanguinity and hereditary risk factors | Not a systematic review. |
| 37 | Bennett, C. M., et al. | 2015 | Lifestyle factors and small intestine adenocarcinoma risk: A systematic review and meta-analysis | Not processed meat. |
| 38 | Berciano, S. and J. M. Ordovas | 2014 | Nutrition and cardiovascular health | Not a systematic review. |
| 39 | Bernstein, A. M. and W. C. Willett | 2011 | Red Meat Intake and the Risk of Cardiovascular Disease | Not a systematic review. |
| 40 | Bertuccio, P., et al. | 2013 | Dietary patterns and gastric cancer risk: A systematic review and meta-analysis | Not processed meat. |
| 41 | Bhat, S., et al. | 2016 | The relationship between dietary patterns and carotid intima media thickness, as an early biomarker of cardiovascular disease: A systematic review and narrative synthesis | Only abstract. |
| 42 | Bonequi, P., et al. | 2013 | Risk factors for gastric cancer in Latin America: A meta-analysis | No quality assessment. |
| 43 | Burch, E., et al. | 2018 | Dietary intake by food group of individuals with type 2 diabetes mellitus: A systematic review | Wrong population. |
| 44 | Bylsma, L., et al. | 2015 | A review and meta-analysis of red meat consumption and type 2 diabetes | Only a conference abstract. No full text available. |
| 45 | Bylsma, L. C. and Alexander D. | 2016 | A review and meta-analysis of prospective studies of red and processed meat, meat cooking methods, Heme iron, heterocyclic amines and prostate cancer | Conference abstract. Reference to the full text paper Bylsma et al. 2015 (ID 46). |
| 46 | Bylsma, L. C. and Alexander, D. D. | 2015 | A review and meta-analysis of prospective studies of red and processed meat, meat cooking methods, heme iron, heterocyclic amines and prostate cancer | No quality assessment. |
| 47 | Caini, S., et al. | 2016 | Food of animal origin and risk of non-Hodgkin lymphoma and multiple myeloma: A review of the literature and meta-analysis | No quality assessment. |
| 48 | Cappellani, A., et al. | 2012 | Diet and pancreatic cancer: many questions with few certainties | Not a systematic review. |
| 49 | Carr, P. R., et al | 2016 | Meat subtypes and their association with colorectal cancer: Systematic review and meta-analysis | Not processed meat. |
| 50 | Cascella, M., et al. | 2018 | Dissecting the mechanisms and molecules underlying the potential carcinogenicity of red and processed meat in colorectal cancer (CRC): An overview on the current state of knowledge | Not a systematic review. |
| 51 | Castro, C., et al. | 2018 | Modifiable factors and esophageal cancer: a systematic review of published meta-analyses | Not processed meat. |
| 52 | Chan, D. S., et al. | 2011 | Red and processed meat and colorectal cancer incidence: meta-analysis of prospective studies | No quality assessment. |
| 53 | Chen, G. C., et al | 2013 | Red and processed meat consumption and risk of stroke: a meta-analysis of prospective cohort studies | No quality assessment. |
| 54 | Cordain, L., et al. | 2002 | The paradoxical nature of hunter-gatherer diets: meat-based, yet non-atherogenic | Not a systematic review. |
| 55 | Cordle, F | 1986 | The use of epidemiology, scientific data, and regulatory authority to determine risk factors in cancers of some organs of the digestive system. 5. Stomach cancer | Not a systematic review. |
| 56 | Crane, T. E., et al. | 2014 | Dietary intake and ovarian cancer risk: a systematic review | No quality assessment. |
| 57 | Crippa, A., et al. | 2018 | Red and processed meat consumption and risk of bladder cancer: a dose-response meta-analysis of epidemiological studies | No quality assessment. |
| 58 | Cross, A. J. and R. Sinha | 2004 | Meat-related mutagens/carcinogens in the etiology of colorectal cancer | Not a systematic review. |
| 59 | Dagnelie, P. C., et al. | 2004 | Diet, anthropometric measures and prostate cancer risk: a review of prospective cohort and intervention studies | No quality assessment. |
| 60 | De Ceglie, A., et al. | 2011 | Barrett's esophagus, esophageal and esophagogastric junction adenocarcinomas: the role of diet | No quality assessment. |
| 61 | Defago, M., et al | 2013 | Association between food patterns and biomarkers of endothelial function: A systematic review. | Not processed meat. |
| 62 | Derbyshire, E. J. and C. H. S. Ruxton | 2015 | Red meat consumption and type 2 diabetes: A systematic review of the evidence | Abstract (summer meeting paper; not a full article). |
| 63 | De Smet, S. and Vossen, E. | 2016 | Meat: The balance between nutrition and health. A review | Not a systematic review. |
| 64 | Domingo, J. L. and Nadal, M. | 2017 | Carcinogenicity of consumption of red meat and processed meat: A review of scientific news since the IARC decision | No quality assessment. |
| 65 | Donaldson, M. S. | 2004 | Nutrition and cancer: A review of the evidence for an anti-cancer diet. | Not a systematic review. |
| 66 | Doyle, V. C | 2007 | Nutrition and colorectal cancer risk: a literature review | Not a systematic review. |
| 67 | Esposito | 2010 | Prevention of type 2 diabetes by dietary patterns: a systematic review of prospective studies and meta-analysis | Not processed meat. |
| 68 | Fallahzadeh, H., et al. | 2014 | Red meat intake and risk of non-Hodgkin lymphoma: a meta-analysis. | No quality assessment. |
| 69 | Fankhauser, C. D. and Mostafid, H. | 2018 | Prevention of bladder cancer incidence and recurrence: Nutrition and lifestyle | Not processed meat. |
| 70 | Faramawi, M. F., et al | 2007 | Consumption of different types of meat and the risk of renal cancer: Meta-analysis of case-control studies | No quality assessment. |
| 71 | Fardet, A., et al. | 2017 | Do alcoholic beverages, obesity and other nutritional factors modify the risk of familial colorectal cancer? A systematic review | No quality assessment. |
| 72 | Feskens, E. J. M., et al. | 2013 | Meat consumption, diabetes, and its complications. | Not a systematic review. |
| 73 | Foroughi, M., et al | 2013 | Stroke and nutrition: A review of studies | No quality assessment. |
| 74 | Gathirua-Mwangi, W. G. and J. Zhang | 2014 | Dietary factors and risk for advanced prostate cancer | No quality assessment. |
| 75 | Gerber, B., et al. | 2003 | Nutrition and lifestyle factors on the risk of developing breast cancer | Not a systematic review. |
| 76 | Gonzales, J. F., et al. | 2014 | Applying the precautionary principle to nutrition and cancer | Not a systematic review. |
| 77 | Gonzalez, C. A., et al. | 2013 | Gastric cancer: epidemiologic aspects | Not a systematic review. |
| 78 | Green, R., et al | 2016 | Dietary patterns in India: A systematic review | Not processed meat. |
| 79 | Grosso, G., et al. | 2013 | Mediterranean diet and cancer: epidemiological evidence and mechanism of selected aspects | Not a systematic review. |
| 80 | Grosso, G., et al. | 2017 | Health risk factors associated with meat, fruit and vegetable consumption in cohort studies: A comprehensive meta-analysis | Wrong exposure & outcome. |
| 81 | Guo, J., et al. | 2015 | Red and processed meat intake and risk of breast cancer: a meta-analysis of prospective studies | No quality assessment.  Authors use Newcastle-Ottowa scale, but do not report the result for each study. |
| 82 | Hammerling, U., et al | 2016 | Consumption of Red/Processed Meat and Colorectal Carcinoma: Possible Mechanisms Underlying the Significant Association | Not a systematic review. |
| 83 | Hariharan, D., et al. | 2015 | The Western Diet and Chronic Kidney Disease. | Not a systematic review. |
| 84 | Hart, A. R., et al. | 2008 | Pancreatic cancer: a review of the evidence on causation | No quality assessment. |
| 85 | Hjartaker, A. | 2003 | Fish consumption and risk of breast, colorectal and prostate cancer: A critical evaluation of epidemiological studies | Not a systematic review. |
| 86 | Hoffman, R. and M. Gerber | 2015 | Food Processing and the Mediterranean Diet | Not a systematic review. |
| 87 | Hori, S., et al. | 2011 | Prostate cancer and diet: food for thought? | No quality assessment.  Priority in regards to study design, but no quality assessment is given for each study. |
| 88 | Huang | 2013 | Red and processed meat intake and risk of esophageal adenocarcinoma: a meta-analysis of observational studies | No quality assessment. |
| 89 | Huncharek | 2003 | Dietary cured meat and the risk of adult glioma: a meta-analysis of nine observational studies | No quality assessment. |
| 90 | Huxley, R. R., et al. | 2009 | The impact of dietary and lifestyle risk factors on risk of colorectal cancer: a quantitative overview of the epidemiological evidence | No quality assessment. |
| 91 | Jacobs, D. R., Jr. and L. C. Tapsell | 2015 | What an anticardiovascular diet should be in 2015 | Not a systematic review. |
| 92 | Jakszyn, P. and C. A. Gonzalez | 2006 | Nitrosamine and related food intake and gastric and oesophageal cancer risk: a systematic review of the epidemiological evidence | No quality assessment. |
| 93 | Jankovic, N. | 2015 | Adherence to the WCRF/AICR dietary recommendations for cancer prevention and risk of cancer in elderly, the chances consortium | Not a systematic review. |
| 94 | Jankovic, N., et al. | 2017 | Adherence to the WCRF/AICR Dietary Recommendations for Cancer Prevention and Risk of Cancer in Elderly from Europe and the United States: A Meta-Analysis within the CHANCES Project | Not processed meat. |
| 95 | Jannasch, F., et al. | 2016 | Dietary patterns and Type 2 Diabetes-systematic review and meta-analysis | Only conference abstract (annual meeting). |
| 96 | Jannasch, F., et al. | 2017 | Dietary patterns and Type 2 diabetes: A systematic literature review and meta-analysis of prospective studies | Not processed meat. |
| 97 | Johnson, C. M., et al. | 2013 | Meta-analyses of colorectal cancer risk factors | No quality assessment. |
| 98 | Johnson, I. T. and E. K. Lund | 2007 | Review article: nutrition, obesity and colorectal cancer | No quality assessment. |
| 99 | Jun, S., et al. | 2016 | Meat and milk intake in the rice-based Korean diet: Impact on cancer and metabolic syndrome | Not a systematic review. |
| 100 | Kaluza, J., et al. | 2012 | Red meat consumption and risk of stroke: a meta-analysis of prospective studies | No quality assessment. |
| 101 | Kantha, S. S. | 1990 | Nutrition and health in China, 1949 to 1989 | Not a systematic review. |
| 102 | Key, T. J., et al. | 2004 | Diet, nutrition and the prevention of cancer | Not a systematic review. |
| 103 | Kim, E., et al. | 2013 | Review of the association between meat consumption and risk of colorectal cancer | Not a systematic review. |
| 104 | Kim, K., et al. | 2017 | Total, red, processed, and white meat intake and stroke incidence and mortality: A systematic review and meta-analysis of cohort studies | Conference abstract. |
| 105 | Kim, Y., et al. | 2015 | A review of potential metabolic etiologies of the observed association between red meat consumption and development of type 2 diabetes mellitus | Not a systematic review. |
| 106 | Kim, Y. and Je, Y. | 2018 | Meat consumption and risk of metabolic syndrome: Results from the Korean population and a meta-analysis of observational studies | Wrong outcome. |
| 107 | Knize, M. G. and J. S. Felton | 2005 | Formation and human risk of carcinogenic heterocyclic amines formed from natural precursors in meat | Not a systematic review. |
| 108 | Kolahdooz, F., et al. | 2010 | Meat, fish, and ovarian cancer risk: Results from 2 Australian case-control studies, a systematic review, and meta-analysis | No quality assessment. |
| 109 | Kontogianni, M. D. and D. B. Panagiotakos | 2007 | Current epidemiological and clinical evidence on the relationship between Mediterranean diet and the metabolic syndrome | Not a systematic review. |
| 110 | Kotepui, M | 2016 | Diet and risk of breast cancer | Not a systematic review. |
| 111 | Koutsokera, A., et al. | 2013 | Nutrition habits, physical activity, and lung cancer: an authoritative review. | No quality assessment. |
| 112 | Kouvari, M., et al. | 2016 | Red meat consumption and healthy ageing: A review | No quality assessment. |
| 113 | Kouvari, M., et al. | 2016 | Diabetes mellitus associated with processed and unprocessed read meat: an overview | No quality assessment. |
| 114 | Kris-Etherton, P. M., et al. | 2002 | Recent discoveries in inclusive food-based approaches and dietary patterns for reduction in risk for cardiovascular disease | Not a systematic review. |
| 115 | Kubo, A., et al. | 2010 | Dietary factors and the risks of oesophageal adenocarcinoma and Barrett's oesophagus | No quality assessment. |
| 116 | La Vecchia | 1988 | Epidemiological Aspects of Diet and Cancer: A summary review of Case-Control studies | Not a systematic review. |
| 117 | La Vecchia | 2001 | Diet and cancer prevention: A review of Italian studies | Not a systematic review. |
| 118 | Lam, W. K | 2005 | Lung cancer in Asian women-the environment and genes | Not a systematic review. |
| 119 | Lambrinoudaki, I., et al. | 2013 | EMAS position statement: Diet and health in midlife and beyond | Not a systematic review. |
| 120 | Lara, J., et al. | 2014 | Effectiveness of dietary interventions among adults of retirement age: a systematic review and meta-analysis of randomized controlled trials. | Not processed meat. |
| 121 | Larsson, S. C. and N. Orsini | 2014 | Red meat and processed meat consumption and all-cause mortality: a meta-analysis | No quality assessment. |
| 122 | Larsson, S. C., et al. | 2006 | Processed meat consumption and stomach cancer risk: a meta-analysis. | No quality assessment. |
| 123 | Larsson, S. C. and A. Wolk | 2006 | Meat consumption and risk of colorectal cancer: a meta-analysis of prospective studies | No quality assessment. |
| 124 | Larsson, S. C. and A. Wolk | 2012 | Red and processed meat consumption and risk of pancreatic cancer: meta-analysis of prospective studies | No quality assessment |
| 125 | Latino-Martel, P., et al. | 2016 | Alcoholic beverages, obesity, physical activity and other nutritional factors, and cancer risk: A review of the evidence | No quality assessment. |
| 126 | Lee | 1993 | Diet and cancer: a short review | Not a systematic review. |
| 127 | Lee, H., et al. | 2017 | Gender perspectives on the relationship between red and processed meat intake and colorectal cancer: A systematic review and meta-analysis | Conference abstract. |
| 128 | Leitzmann, M. F. and S. Rohrmann | 2012 | Risk factors for the onset of prostatic cancer: Age, location, and behavioral correlates | Not a systematic review. |
| 129 | Li, F., et al. | 2014 | Red and processed meat intake and risk of bladder cancer: A meta-analysis. | No quality assessment. |
| 130 | Lindeberg, S. | 2012 | Paleolithic diets as a model for prevention and treatment of Western disease | Not a systematic review. |
| 131 | Lindeberg, S., et al. | 2003 | Biological and Clinical Potential of a Palaeolithic Diet | Not a systematic review. |
| 132 | Lindgren, J. A., et al. | 2013 | Is Usual Dietary Pattern Related to the Risk of Developing Breast Cancer? | No quality assessment. |
| 133 | Lippi, G., et al. | 2015 | Red meat, processed meat and the risk of venous thromboembolism: friend or foe? | No quality assessment. |
| 134 | Lippi, G., et al. | 2016 | Meat consumption and cancer risk: a critical review of published meta-analyses | No quality assessment. |
| 135 | Lippi, G., et al. | 2015 | Red meat consumption and ischemic heart disease. A systematic literature review | No quality assessment. |
| 136 | Liu, X. D., et al. | 2013 | Meta-analysis of dietary patterns and esophageal cancer risk | Conference abstract. No full text available |
| 137 | Liu, Z. T. and A. H. Lin | 2014 | Dietary factors and thyroid cancer risk: a meta-analysis of observational studies | Not processed meat. |
| 138 | Lochner, J., et al. | 2006 | How effective are lifestyle changes for controlling hypertension? | Not a systematic review. |
| 139 | Lovegrove, C., et al | 2015 | Systematic review of prostate cancer risk and association with consumption of fish and fish-oils: analysis of 495,321 participants | No quality assessment. |
| 140 | Ma, R. W. and K. Chapman | 2009 | A systematic review of the effect of diet in prostate cancer prevention and treatment. | No quality assessment. |
| 141 | Magalhaes, B., et al. | 2012 | Dietary patterns and colorectal cancer: systematic review and meta-analysis. | No quality assessment. |
| 142 | Maghsoudi, Z. and L. Azadbakht | 2012 | How dietary patterns could have a role in prevention, progression, or management of diabetes mellitus? Review on the current evidence | No quality assessment. |
| 143 | Mandair, D., et al. | 2014 | Prostate cancer and the influence of dietary factors and supplements: A systematic review | No quality assessment.  State that the quality and level of evidence of the results were considered, but no quality assessment is given for each study. |
| 144 | Marques-Vidal, P., et al. | 2006 | Foodstuffs and colorectal cancer risk: A review | Not a systematic review. |
| 145 | Martinez-Gonzalez, M. A. and N. Martin-Calvo | 2013 | The major European dietary patterns and metabolic syndrome | No quality assessment. |
| 146 | Matos, E. and A. Brandani | 2002 | Review on meat consumption and cancer in South America | Not a systematic review. |
| 147 | McAfee, A. J., et al. | 2010 | Red meat consumption: an overview of the risks and benefits | Not a systematic review. |
| 148 | McEvoy, C., et al | 2013 | A systematic review and meta-analysis examining 'a posteriori' dietary patterns and risk of type 2 diabetes. | Conference abstract. |
| 149 | McEvoy, C. T., et al. | 2014 | A posteriori dietary patterns are related to risk of type 2 diabetes: findings from a systematic review and meta-analysis. | No quality assessment. |
| 150 | McEvoy, C. T., et al. | 2012 | Vegetarian diets, low-meat diets and health: a review | No quality assessment. |
| 151 | McIntosh, G. H. | 2011 | Diet/lifestyle factors for reducing colorectal cancer risk | Only a conference abstract. No full text available. |
| 152 | McManus, A., et al. | 2011 | Health benefits of seafood for men | Not processed meat. |
| 153 | McNeill, S. and M. E. Van Elswyk | 2012 | Red meat in global nutrition | Not a systematic review. |
| 154 | Micha, R., et al | 2012 | Unprocessed red and processed meats and risk of coronary artery disease and type 2 diabetes - An updated review of the evidence | Not a systematic review. |
| 155 | Miller, P. E. and D. Alexander | 2016 | A review and meta-analysis of prospective studies of red and processed meat and pancreatic cancer | Only abstract. No full article. |
| 156 | Miller, P. E., et al. | 2010 | Dietary patterns and colorectal adenoma and cancer risk: a review of the epidemiological evidence | No quality assessment. |
| 157 | Misra, A., et al. | 2011 | Nutrition transition in India: secular trends in dietary intake and their relationship to diet-related non-communicable diseases | No quality assessment. |
| 158 | Miura, K. and H. Nakagawa | 2005 | Can dietary changes reduce blood pressure in the long term? | Not a systematic review. |
| 159 | Mosby, T. T., et al. | 2012 | Nutrition in adult and childhood cancer: role of carcinogens and anti-carcinogens | Not a systematic review. |
| 160 | Mourouti, N., et al. | 2015 | Diet and breast cancer: a systematic review | No quality assessment. |
| 161 | Murakami | 2005 | Effect of dietary factors on incidence of type 2 diabetes: A systematic review of cohort studies | No quality assessment. |
| 162 | Muscaritoli, M., et al. | 2016 | Foods and their components promoting gastrointestinal cancer | Not a systematic review. |
| 163 | Namiranian, N., et al. | 2014 | Risk factors of breast cancer in the Eastern Mediterranean Region: a systematic review and meta-analysis | Not processed meat. |
| 164 | Ndanuko, R. N., et al | 2016 | Dietary Patterns and Blood Pressure in Adults: A Systematic Review and Meta-Analysis of Randomized Controlled Trials | Not processed meat. |
| 165 | Niclis, C., et al. | 2012 | Dietary habits and prostate cancer prevention: a review of observational studies by focusing on South America | No quality assessment. |
| 166 | Norat, T. and Riboli E. | 2001 | Meat consumption and colorectal cancer: a review of epidemiologic evidence | Not a systematic review. |
| 167 | Norat, T., et al. | 2002 | Meat consumption and colorectal cancer risk: dose-response meta-analysis of epidemiological studies | No quality assessment. |
| 168 | Nothlings, U. | 2012 | Combined lifestyle factors and chronic disease risk | Conference abstract. No full text available. |
| 169 | O'Connor, L. E., et al | 2016 | Consuming < or > 0.5 servings of red meat per day does not have a negative impact on cardiovascular disease risk factors; a systematic review and meta-analysis of randomized controlled trials. | Only abstract. From the Experimental Biology 2016 meeting. |
| 170 | O’Connor, L., et al. | 2017 | Total red meat intake of >=0.5 servings/d does not negatively influence cardiovascular disease risk factors: A systemically searched meta-analysis of randomized controlled trials | Not processed meat. |
| 171 | Paluszkiewicz, P., et al. | 2012 | Main dietary compounds and pancreatic cancer risk. The quantitative analysis of case-control and cohort studies | Not processed meat. |
| 172 | Pan, P., et al. | 2018 | Colon Cancer: What We Eat | No quality assessment. |
| 173 | Pereira, P. M. and A. F. Vicente | 2013 | Meat nutritional composition and nutritive role in the human diet | Not a systematic review. |
| 174 | Petrick, J. L., et al. | 2015 | Dietary Risk Reduction Factors for the Barrett's Esophagus-Esophageal Adenocarcinoma Continuum: A Review of the Recent Literature. | Not a systematic review. |
| 175 | Pham, N. M., et al | 2014 | Meat consumption and colorectal cancer risk: an evaluation based on a systematic review of epidemiologic evidence among the Japanese population. | No quality assessment. |
| 176 | Pighin, D., et al. | 2016 | A Contribution of Beef to Human Health: A Review of the Role of the Animal Production Systems | Not a systematic review. |
| 177 | Ponz de Leon, M. and L. Roncucci | 2000 | The cause of colorectal cancer | Not a systematic review. |
| 178 | Psaltopoulou, T., et al. | 2010 | The role of diet and lifestyle in primary, secondary, and tertiary diabetes prevention: a review of meta-analyses | No quality assessment.  Only RCT, but no quality assessment is given for each study. |
| 179 | Qasim, A. and C. O'Morain | 2010 | Primary prevention of colorectal cancer: are we closer to reality? | Not a systematic review. |
| 180 | Qu, X., et al. | 2013 | Consumption of red and processed meat and risk for esophageal squamous cell carcinoma based on a meta-analysis. | No quality assessment. |
| 181 | Randi, G., et al. | 2010 | Dietary patterns and the risk of colorectal cancer and adenomas | No quality assessment. |
| 182 | Rees, K., et al. | 2013 | "mediterranean" dietary pattern for the primary prevention of cardiovascular disease: A cochrane systematic review | Abstract. Full text reference ID 161. |
| 183 | Rees, K., et al. | 2013 | "mediterranean" dietary pattern for the primary prevention of cardiovascular disease: A cochrane systematic review | Not processed meat. |
| 184 | Richi, E. B., et al. | 2015 | Health risks associated with meat consumption: A review of epidemiological studies | Not a systematic review. |
| 185 | Rohrmann, S. and J. Linseisen | 2016 | Processed meat: The real villain? | Not a systematic review. |
| 186 | Rossi, R. E., et al | 2014 | The role of dietary factors in prevention and progression of breast cancer | No quality assessment.  State that the quality and level of evidence was considered, but no quality assessment is given for each study. |
| 187 | Rouhani, M. H., et al | 2014 | Is there a relationship between red or processed meat intake and obesity? A systematic review and meta-analysis of observational studies | No quality assessment. |
| 188 | Ryan-Harshman, M. and W. Aldoori | 2007 | Diet and colorectal cancer: Review of the evidence | No quality assessment.  Level of evidence is provided, however quality assessment within each study is not assessed. |
| 189 | Sadri, G. H. and H. Mahjub | 2006 | Meat consumption is a risk factor for colorectal cancer: Meta-analysis of case-control studies | No quality assessment. |
| 190 | Salas-Salvado, J., et al. | 2011 | The role of diet in the prevention of type 2 diabetes. | Not processed meat. |
| 191 | Salehi, M., et al | 2013 | Meat, fish, and esophageal cancer risk: a systematic review and dose-response meta-analysis | No quality assessment.  Make quality assessment using the Critical Appraisal Skills Programme, but they do not report on the results from this assessment. |
| 192 | Sandhu, M. S., et al | 2001 | Systematic review of the prospective cohort studies on meat consumption and colorectal cancer risk: a meta-analytical approach | No quality assessment. |
| 193 | Santarelli, R. L., et al. | 2008 | Processed meat and colorectal cancer: a review of epidemiologic and experimental evidence. | Not a systematic review. |
| 194 | Saweri | 2001 | The rocky road from roots to rice: a review of the changing food and nutrition situation in Papua New Guinea | Not a systematic review. |
| 195 | Schatzkin, A. and G. Kelloff | 1995 | Chemo- and dietary prevention of colorectal cancer | Not a systematic review. |
| 196 | Schulz, M., et al. | 2004 | Dietary determinants of epithelial ovarian cancer: a review of the epidemiologic literature | No quality assessment. |
| 197 | Schwingshackl, L. and G. Hoffmann | 2016 | Does a Mediterranean-Type Diet Reduce Cancer Risk | Not a systematic review. |
| 198 | Schwingshackl, L., et al. | 2017 | Food groups and risk of all-cause mortality: A systematic review and meta-analysis of prospective studies | Wrong outcome. |
| 199 | Schwingshackl, L., et al. | 2017 | Food groups and risk of type 2 diabetes mellitus: a systematic review and meta-analysis of prospective studies | No quality assessment.  The authors indicate that they did risk of bias assessment, but they do not provide the report the result for each study. |
| 200 | Schwingshackl, L., et al. | 2017 | Food Groups and Risk of Hypertension: A Systematic Review and Dose-Response Meta-Analysis of Prospective Studies | Wrong outcome. |
| 201 | Schwingshackl, L., et al. | 2018 | Food groups and risk of colorectal cancer | No quality assessment.  The authors indicate that they did risk of bias assessment, but they do not provide the report the result for each study. |
| 202 | Serra-Majem, L., et al. | 1995 | How could changes in diet explain changes in coronary heart disease mortality in Spain? The Spanish paradox | Not a systematic review. |
| 203 | Shen, J., et al. | 2015 | Mediterranean Dietary Patterns and Cardiovascular Health | Not a systematic review. |
| 204 | Sian | 1987 | Diet and nutritional factors in the aetiology of colon cancer (review) | Not a systematic review. |
| 205 | Skibola, C. F. | 2007 | Obesity, diet and risk of non-Hodgkin lymphoma | Not a systematic review. |
| 206 | Smolinska, K. and P. Paluszkiewicz | 2010 | Risk of colorectal cancer in relation to frequency and total amount of red meat consumption. Systematic review and meta-analysis | Not processed meat. |
| 207 | Sofi, F., et al | 2013 | Mediterranean diet and health | Not a systematic review. |
| 208 | Song, P., et al. | 2014 | Red meat consumption and stomach cancer risk: a meta-analysis | Not processed meat. |
| 209 | Steinmaus, C. M., et al | 2000 | Diet and bladder cancer: a meta-analysis of six dietary variables. | No quality assessment. |
| 210 | Stepien, M. et al. | 2016 | The role of diet in cancer: the epidemiologic link | Not a systematic review. |
| 211 | Stettler, N., et al. | 2013 | Systematic review of clinical studies related to pork intake and metabolic syndrome or its components | No quality assessment. |
| 212 | Stoody, E., et al. | 2014 | Dietary patterns and risk of cardiovascular disease: A systematic review | Conference abstract. No full text available. |
| 213 | Tabung, F. K., et al. | 2017 | Dietary Patterns and Colorectal Cancer Risk: a Review of 17 Years of Evidence (2000-2016) | Not processed meat. |
| 214 | Tahbaz, R., et al. | 2018 | Prevention of kidney cancer incidence and recurrence: Lifestyle, medication and nutrition | Not processed meat. |
| 215 | Tang, Y., et al | 2011 | Nitrite and nitrate: cardiovascular risk-benefit and metabolic effect | Not a systematic review. |
| 216 | Tarraga Lopez, P. J., et al | 2014 | Primary and secondary prevention of colorectal cancer | No quality assessment. |
| 217 | Taylor, V. H., et al. | 2009 | Is red meat intake a risk factor for breast cancer among premenopausal women? | No quality assessment. |
| 218 | Tian, S., et al. | 2017 | Dietary protein consumption and the risk of type 2 diabetes: A systematic review and meta-analysis of cohort studies | No quality assessment. |
| 219 | Togo, P., et al | 2001 | Food intake patterns and body mass index in observational studies | No quality assessment. |
| 220 | Tsugane, S. and S. Sasazuki | 2007 | Diet and the risk of gastric cancer: review of epidemiological evidence | Not a systematic review. |
| 221 | Van Meer, S., et al. | 2013 | Role of dietary factors in survival and mortality in colorectal cancer: a systematic review | No quality assessment. |
| 222 | Vargas, A. J. and P. A. Thompson | 2012 | Diet and nutrient factors in colorectal cancer risk | Not a systematic review. |
| 223 | Vieira, A., et al. | 2017 | Foods and beverages and colorectal cancer risk: A systematic review and meta-analysis of cohort studies, an update of the evidence of the WCRF-AICR Continuous Update Project | No quality assessment. |
| 224 | Wang, C. and H. Jiang | 2012 | Meat intake and risk of bladder cancer: a meta-analysis | No quality assessment. |
| 225 | Wang, Q., et al. | 2014 | The Mediterranean diet and gastrointestinal cancers risk | Not a systematic review. |
| 226 | Wei, Y., et al. | 2015 | Association between processed meat and red meat consumption and risk for glioma: a meta-analysis from 14 articles | No quality assessment. |
| 227 | Weisbeck, A. and Jansen, R. J. | 2017 | Nutrients and the pancreas: An epigenetic perspective | Not a systematic review. |
| 228 | Weiss, J. R., et al. | 2005 | Epidemiology of male breast cancer. | Not a systematic review. |
| 229 | White, D. L. and A. Collinson | 2013 | Red meat, dietary heme iron, and risk of type 2 diabetes: the involvement of advanced lipoxidation endproducts | Not a systematic review. |
| 230 | Whiteman, D. C. and L. F. Wilson | 2016 | The fractions of cancer attributable to modifiable factors: A global review | No quality assessment. |
| 231 | Wigle, D. T., et al. | 2008 | Role of hormonal and other factors in human prostate cancer. | Not a systematic review. |
| 232 | Willett, W. C. | 1995 | Diet, nutrition, and avoidable cancer | Not a systematic review. |
| 233 | Woo, H. D., et al. | 2014 | Diet and cancer risk in the Korean population: a meta- analysis | No quality assessment. |
| 234 | Wunsch-Filho, V. and E. A. de Camargo | 2001 | The burden of mouth cancer in Latin America and the Caribbean: epidemiologic issues | Not a systematic review. |
| 235 | Xu, B., et al. | 2013 | No evidence of decreased risk of colorectal adenomas with white meat, poultry, and fish intake: a meta-analysis of observational studies | No quality assessment. |
| 236 | Xu, X., et al. | 2013 | Red and processed meat intake and risk of colorectal adenomas: A meta-analysis of observational studie | No quality assessment. |
| 237 | Xue, X. J., et al. | 2014 | Red and processed meat consumption and the risk of lung cancer: A dose-response meta-analysis of 33 published studies. | No quality assessment. |
| 238 | Yang, C., et al | 2016 | Red Meat Consumption and the Risk of Stroke: A Dose-Response Meta-analysis of Prospective Cohort Studies | Insufficient literature search.  Mention two databases in abstract (PubMed, Embase), but only one is presented in method section and flow chart (PubMed), and they do not mention removal of duplicates. |
| 239 | Yang, F., et al. | 2016 | Association between processed meat and red meat consumption and risk of nasopharyngeal carcinoma: Evidence from a meta-analysis | No quality assessment.  In table 2 a quality score is presented, but it is unclear which quality assessment tool the evaluation is based on. |
| 240 | Yoon, H., et al. | 2000 | Systematic review of epidemiological studies on meat, dairy products and egg consumption and risk of colorectal adenomas | Wrong outcome. |
| 241 | Yusof, A. S., et al. | 2012 | Dietary patterns and risk of colorectal cancer: a systematic review of cohort studies (2000-2011) | No quality assessment. |
| 242 | Zandonai, A. P., et al. | 2012 | The dietary risk factors for colorectal neoplasms focus in meat | No quality assessment.  Results from the quality assessment using Jadad Scale is not shown. |
| 243 | Zheng, W. and S. A. Lee | 2009 | Well-done meat intake, heterocyclic amine exposure, and cancer risk | No quality assessment. |
| 244 | Zhou, S. S., et al. | 2011 | Dietary methyl-consuming compounds and metabolic syndrome | Not a systematic review. |
