# Supplementary material for: Processed meat intake and chronic disease morbidity and mortality: An overview of systematic reviews and meta-analyses
Source: PLoS One. 2019 Oct 17;14(10):e0223883. doi: 10.1371/journal.pone.0223883 (PMC6797176; doi:10.1371/journal.pone.0223883)
Supplement: S3 Table — (DOCX) [file pone.0223883.s003.docx]

**Supplemental table 3.** Quality assessment using AMSTAR.

| **Review** | **Q1** | **Q2** | **Q3** | **Q4** | **Q5** | **Q6** | **Q7** | **Q8** | **Q9** | **Q10** | **Q11** | **Final score** |
| --- | --- | --- | --- | --- | --- | --- | --- | --- | --- | --- | --- | --- |
| Choi (2013) | No | Yes | Yes | No | No | Yes | Yes | No | Yes | Yes | No | 6 |
| Fang (2015) | No | Yes | Yes | No | No | Yes | Yes | No | Yes | Yes | No | 6 |
| Kim (2017) | No | Yes | Yes | No | No | Yes | Yes | No | Yes | Yes | No | 6 |
| Li (2012) | No | Yes | Yes | Yes | No | No | Yes | Yes | N/A | N/A | Yes | 6 |
| Li (2016) | No | No | Yes | No | No | Yes | Yes | No | Yes | Yes | No | 5 |
| Luo (2014) | No | No | Yes | No | No | Yes | Yes | No | Yes | Yes | No | 5 |
| Micha (2010) | No | No | Yes | Yes | Yes | Yes | Yes | No | Yes | Yes | No | 7 |
| O´Sulivan (2013) | No | Yes | Yes | No | No | Yes | Yes | Yes | Yes | Yes | No | 7 |
| Quach (2016) | No | Yes | Yes | Yes | Yes | No | Yes | Yes | N/A | N/A | Yes | 7 |
| Saneei (2015) | Yes | Yes | Yes | No | Yes | Yes | Yes | No | Yes | Yes | No | 8 |
| Solimini (2016) | No | Yes | Yes | No | Yes | Yes | Yes | No | Yes | Yes | No | 7 |
| Wallin (2011) | No | No | Yes | No | Yes | Yes | Yes | No | Yes | Yes | No | 6 |
| Wang (2016) | No | Yes | Yes | No | Yes | Yes | Yes | No | Yes | Yes | No | 7 |
| Xu (2014) | No | Yes | Yes | No | Yes | Yes | Yes | No | Yes | Yes | No | 7 |
| Yang (2012) | No | Yes | Yes | No | No | Yes | Yes | Yes | Yes | Yes | No | 7 |
| Yang (2015) | No | Yes | Yes | No | No | Yes | Yes | Yes | Yes | Yes | No | 7 |
| Zhang (2017) | No | Yes | Yes | No | No | Yes | Yes | No | Yes | Yes | No | 6 |
| Zhao (2017), CRC | No | Yes | Yes | No | No | Yes | Yes | No | Yes | Yes | No | 6 |
| Zhao (2017), PC | No | Unclear | Yes | No | No | Yes | Yes | No | Yes | Yes | No | 5 |
| Zhao (2017), GC | No | Yes | Yes | No | No | Yes | Yes | No | Yes | Yes | No | 6 |
| Zhu (2013) | No | Yes | Yes | No | Yes | Yes | Yes | No | Yes | Yes | No | 7 |
| Zhu (2014) | No | Yes | Yes | No | No | Yes | Yes | No | Yes | Yes | No | 6 |
| *Post hoc quality assessment* |  |  |  |  |  |  |  |  |  |  |  |  |
| Yang (2016) | No | Yes | No | Yes | No | Yes | Yes | No | Yes | Yes | No | 6 |
| Total, k=23 | N: k=22  Y: k=1 | N: k=4  Y: k=18  U: k=1 | N: k=1  Y: k=22 | N: k=19  Y: k=4 | N: k=15  Y: k=8 | N: k=2  Y: k=21 | N: k= 0  Y: k=23 | N: k=18  Y: k=5 | N: k=0  Y: k=21  N/A: k=2 | N: k=0  Y: k=21  N/A: k=2 | N: k=21  Y: k=2 |  |

AMSTAR items: Q1. Was an a priori design provided? Q2. Was there duplicate study selection and data extraction? Q3. Was a comprehensive literature search performed? Q4. Was the status of publication (i.e. grey literature) used an inclusion criterion? Q5. Was a list of studies (included and excluded) provided? Q6. Were the characteristics of the included studies provided? Q7. Was the scientific quality of the included studies assessed and documented? Q8. Was the scientific quality of the included studies used appropriately in formulating conclusions? Q9. Were the methods used to combine the findings of studies appropriate? Q10. Was the likelihood of publication bias assessed? Q11. Was the conflict of interest stated? CRC: colorectal cancer; PC: pancreatic cancer; GC: gastric cancer.
