# Supplementary material for: Processed meat intake and chronic disease morbidity and mortality: An overview of systematic reviews and meta-analyses
Source: PLoS One. 2019 Oct 17;14(10):e0223883. doi: 10.1371/journal.pone.0223883 (PMC6797176; doi:10.1371/journal.pone.0223883)
Supplement: S4 Table — Matrix showing the studies (left column) that the estimates (top row) are based on. (DOCX) [file pone.0223883.s004.docx]

**Supplemental 4:** Study Matrix. Matrix showing the studies (left column) that the estimates (top row) are based on.

| **Esophageal cancer** |  | |
| --- | --- | --- |
| **Primary research articles included in review articles** | **Included systematic reviews** | |
| **First author, year, and country** | Choi (2013) | Zhu (2014) |
| Case-control studies |  |  |
| Bosetti (2000), Italy | x | x |
| Chen (2002), USA | x | x |
| Chen (2009), Taiwan | x |  |
| De Stefani (1990), Uruguay |  | x |
| De Stefani (1999), Uruguay |  | x |
| De Stefani (2012), Uruguay | x | x |
| Hajizadh (2011), Iran | x | x |
| Levi (2004), Switzerland | x | x |
| Li (2003), China | x |  |
| O´Doherty (2011), Ireland | x | x |
| Sapkota (2008), Europe | x | x |
| Takezaki (2001), China | x | x |
| Tavani (1994), Italy | x |  |
| Ward (1997), USA |  | x |
| Ward (2012), USA | x |  |
| Wu (2007), USA | x | x |
| Yang (2005), China | x |  |
| Yu (1988), USA | x |  |
| Cohort studies |  |  |
| Cross (2011), USA | x | x |
| González (2006), Europe | x | x |
| Keszei (2012), Netherlands | x | x |

| **Nasopharyngeal carcinoma** |  |  |  |
| --- | --- | --- | --- |
| **Primary research articles included in review articles** | **Included systematic reviews** |  |  |
| **First author, year, and country** | Li (2016) |  |  |
| Case-control studies |  |  |  |
| Armstrong (1983), Malaysia (ethnic Chinese) | x |  |  |
| Armstrong (1998), China | x |  |  |
| Chelleng (2000), India | x |  |  |
| Feng (2007), African (multicenter) | x |  |  |
| Jeannel (1990), Tunisia | x |  |  |
| Jia (2010), China | x |  |  |
| Lorembam (2015), India | x |  |  |
| Luo (2009), China | x |  |  |
| Polesel (2013), Italy | x |  |  |
| Xu (2010), China | x |  |  |
| Yu (1986), Hong Kong | x |  |  |
| Yu (1988), China | x |  |  |
| Zou (2003), China | x |  |  |
|  |  |  |  |
| **Pancreatic cancer** |  | |  |
| **Primary research articles included in review articles** | **Included systematic reviews** | | |
| **First author, year, and country** | Zhao (2017) | | |
| Case-control studies |  | |  |
| La Vecchia (1990), Italy  Ghadirian (1995), Canada  Chan (2007), USA  Polesel (2010), Italy  Ghadirian (2010), Canada  Liu (2014), China | x  x  x  x  x  x | |  |
| Cohort studies |  | |  |
| Zheng (1993), USA  Stolzenberg-Solomon (2002), Finland  Michaud (2003), USA  Nothlings (2005), USA  Lin (2006), Japan  Larsson (2006), Sweden  Stolzenberg-Solomon (2007), USA  Heinen (2009), Netherlands  Aschebrook-Kolfoy (2011), USA  Anderson (2012), USA  Rohormann (2013), Europe  Jiao (2015), USA  Ghorbani (2016), Iran  Taunk (2016), USA | x  x  x  x  x  x  x  x  x  x  x  x  x  x | |  |

| **Hepatocellular carcinoma** |  |
| --- | --- |
| **Primary research articles included in review articles** | **Included systematic reviews** |
| **First author, year, and country** | Luo (2014) |
| Case-control studies |  |
| Kanazir (2010), Serbia | x |
| Talamini (2006), Italy | x |
| Cohort studies |  |
| Cross (2007), USA | x |
| Fedirko (2013), Europe, | x |
| Kurozawa (2004), Japan | x |

| **Gastric cancer** |  | | |  |
| --- | --- | --- | --- | --- |
| **Primary research articles included in review articles** | **Included systematic reviews** | | |  |
| **First author, year, and country** | Fang (2015) | Li (2012) | Zhu (2013) | Zhao (2017)§ |
| Case-control studies |  |  |  |  |
| Aune (2009), Uruguay |  |  | x |  |
| Boeing (1991), Europe |  |  | x |  |
| De Stefani (2004), Uruguay |  |  | x |  |
| González (1991), Spain |  |  | x | x |
| Hoshiyama (1992), Japan |  |  | x |  |
| Hu (2008), USA (Japanese ancestry) |  |  | x |  |
| Ito (2003), Japan |  |  | x | x |
| Lee (1990), Taiwan |  |  | x |  |
| Nomura (2003), USA |  |  | x |  |
| Palli (2001), Italy |  |  | x |  |
| Phukan (2006), India |  |  | x | x |
| Pourfarzi (2009), Iran |  |  | x | x |
| Strumylaité (2006), Lithuania |  |  | x | x |
| Takezaki (2001), China |  |  | x |  |
| Ward (1997), USA |  |  | x |  |
| Ward (1999), USA |  |  | x |  |
| Wu (2007), USA  Risch (1985), Canada  La Vecchia (1987), Italy  Lee (1990), China  Boeing (1991), Germany  Boeing (1991), Poland  Sanchez-Diez (1992), Spain  Hoshiyama (1992), Japan  Nazario (1993), Puerto Rico  Hansson (1993), Sweden  Munoz (1997), Italy  Ward (1999), Mexico  Palli (2001), Italy  Chen (2002), USA  Lissowska (2004), Poland  Gao (2011), China  Hu (2011), Canada  Ward (2012), USA  De Stefani (2012), Uruguay  Lin (2014), China |  |  | x | x  x  x  x  x  x  x  x  x  x  x  x  x  x  x  x  x  x  x  x |
| Cohort studies |  |  |  |  |
| Cross (2011), USA | x |  | x |  |
| Galanis (1998), Japan | x |  | x |  |
| González (2006), Europe | x |  | x | x |
| Keszei (2012), Netherlands | x |  | x | x |
| Knekt (1999), Finland |  |  | x | x |
| Khan (2004), Japan | x |  |  |  |
| Kneller (1991), USA | x |  |  |  |
| Larsson (2006), Sweden | x |  | x | x |
| McCullough (2001), USA | x |  | x |  |
| Ngoan (2002), Japan | x |  | x |  |
| Nomura (1990), USA (Japanese ancestry) | x |  | x | x |
| Tokui (2005), Japan | x |  |  |  |
| Van den Brandt (2003), Netherlands  Zheng (1995), USA  Galanis (1998), USA  Cross (2007), USA  Cross (2011)¤ | x |  |  | x  x  x  x |
| Systematic Reviews |  |  |  |  |
| Jakszyn (2006), Spain* |  | x |  |  |
| Larsson (2006), Sweden* |  | x |  |  |
| *Not included in our systematic review: no quality assessment | | | |  |

§ Did not provide a reference list of included studies, thus in some instances it is difficult to be certain if there are additional common primary studies between the reviews.

¤ Unclear if it is the same study as Cross (2011), USA (the first study listed under cohort studies) or Cross (2007), USA (the study before this one). Since no reference list of the included studies for Zhao et al. was provided, we cannot be certain.

| **Glioma** | |  | |
| --- | --- | --- | --- |
| **Primary research articles included in review articles** | | **Included systematic reviews** | |
| **First author, year, and country** | | Quach (2016) | Saneei (2015) |
| Case-control studies | |  |  |
| Ahlbom (1986), Sweden | |  | x |
| Blowers (1997), USA | |  | x |
| Boeing (1993), Germany | |  | x |
| Burch (1987), Canada | |  | x |
| Chen (2002), USA | |  | x |
| Giles (1994), Australia | |  | x |
| Hochberg (1990), USA | |  | x |
| Hu (2008), Canada | |  | x |
| Kaplan (1997), Israel | |  | x |
| Lee (1997), USA | |  | x |
| Preston-Martin (1991), USA | |  | x |
| Shayanfar (2014), Iran | |  | x |
| Terry (2009), (multicenter: Europe, Northern America, Ausralia) | |  | x |
| Cohort studies | |  |  |
| Dubrow (2010), USA | |  | x |
| Michaud (2009), USA | |  | x |
| Mills (1989), USA | |  | x |
| Rollison (2004), 2004^§^ | |  | x |
| Systematic Reviews | |  |  |
| Huncharek (2003), USA* | | x |  |
| ^§^Nested case-control study  *Not included in our systematic review: no quality assessment | | | |
| **Ovarian cancer** |  | | |
| **Primary research articles included in review articles** | **Included systematic reviews** | | |
| **First author, year, and country** | Wallin (2011) | | |
| Cohort studies |  | | |
| Bertone (2002), USA | x | | |
| Cross (2007), USA | x | | |
| Gilseng (2011), Netherlands | x | | |
| Larsson (2005), Sweden | x | | |
| Schulz (2007), Europe | x | | |

| **Non-Hodgkin lymphoma** |  | |
| --- | --- | --- |
| **Primary research articles included in review articles** | **Included systematic reviews** | |
| **First author, year, and country** | Solimini (2016) | Yang (2015) |
| Case-control studies |  |  |
| Aschebrook-Kilfoy (2012), USA | x | x |
| Chang (2005), Sweden |  | x |
| Charbonneau (2013), USA | x | x |
| Chiu (2008), USA |  | x |
| Cross (2006), USA | x | x |
| De Stefani (1998), Uruguay | x | x |
| De Stefani (2013), Uruguay | x | x |
| Hu (2008), Canada | x | x |
| Hu (2011), Europe |  | x |
| Ollberding (2013), USA |  | x |
| Purdue (2004), Canada |  | x |
| Talamini (2006), Italy | x | x |
| Ward (1994), USA | x |  |
| Zheng (2004), USA |  | x |
| Cohort studies |  |  |
| Chiu (1996), USA | x | x |
| Daniel (2012), USA | x | x |
| Rohrmann (2010), Europe | x | x |

| **Lung cancer** |  |
| --- | --- |
| **Primary research articles included in review articles** | **Included systematic reviews** |
| **First author, year, and country** | Yang (2012) |
| Case-control studies |  |
|  | Not reported |
| Cohort studies |  |
|  | Not reported |

| **Oral cavity and orophanx cancer** |  |
| --- | --- |
| **Primary research articles included in review articles** | **Included systematic reviews** |
| **First author, year, and country** | Xu (2014) |
| Case-control studies |  |
| De Stefani (2012), Uruguay | x |
| Garrote (2001), Cuba | x |
| Levi (2004), Switzerland | x |
| Lissowska (2003), Poland^§^ | x |
| Rajkumar (2003), India | x |
| Sánchez (2003), Spain | x |
| Sapkota (2008), central and eastern Europe | x |
| Toporcov (2004), Brazil | x |
| Zheng (1993), China^¤^ | x |
| Zheng (1992), China | x |
| ^§^Not included in the meta-analysis but are described in the characteristics table  ^¤^Included in the meta-analysis, but is about total meat, not processed meat | |

| **Renal cell carcinoma** |  |  |
| --- | --- | --- |
| **Primary research articles included in review articles** | **Included systematic reviews** | |
| **First author, year, and country** | Zhang (2017) | |
| Case-control studies |  |  |
| De Stefani (2012), Uruguay  Aune (2009), Uruguay  Bravi (2007), Italy  Hsu (2007), Europe  De Stefani (1998), Uruguay  Talamini (1990), Italy  Hu (2011), Canada  Daniel (2011), USA  Brock (2009), USA  Grieb (2009), USA  Hu (2003), Canada  Yuan (1998), USA  Wolk (1996), multicenters  Chow (1994), USA  Maclure (1990), USA | x  x  x  x  x  x  x  x  x  x  x  x  x  x  x |  |
| Cohort studies |  |  |
| Rohrmann (2015), Europe | x |  |
| Daniel (2012), USA | x |  |
| Lee (2008), Europe and USA | x |  |
| Washio (2005), Japan | x |  |

| **Colorectal cancer** |  |  |
| --- | --- | --- |
| **Primary research articles included in review articles** | **Included systematic reviews** | |
| **First author, year, and country** | Zhao (2017) | |
| Case-control studies |  |  |
| Tiemersma(2002), Netherlands  Levi (2004), Switzerland  Kuriki (2006), Japan  Kimura (2007), Japan  Squires (2010), Canada  Spencer (2010), UK  Wang (2010), USA  Williams (2010), USA^1^  De Stefani (2012), Uruguay  Joshi (2015), USA  Miller (2013), USA | x  x  x  x  x  x  x  x  x  x  x |  |
| Cohort studies |  |  |
| Knekt (1999), Finland  Flood (2003), USA  Lin (2004), USA  English (2004), Australia  Larsson (2005), Sweden  Norat (2005), Europe  Sato (2006), Japan  Cross (2007), Usa  Lee (2009), China  Ollberding (2012), USA  Bernstein (2015), USA  Gilsing (2015), Netherlands | x  x  x  x  x  x  x  x  x  x  x  x |  |

^1^ Included in the meta-analysis of colorectal cancer, but the Zhao et al.’s manuscript, namely in supplementary table 4, colon cancer is presented as the only outcome of the primary study from Williams et al. (2010).

| **Colon cancer** |  |  |
| --- | --- | --- |
| **Primary research articles included in review articles** | **Included systematic reviews** | |
| **First author, year, and country** | Zhao (2017) | |
| Case-control studies |  |  |
| Steinmetz (1993), USA  Kampman (1999), USA  Le Marchand (2002), USA  Kimura (2007), Japan  Chiu (2003), China  Spencer (2010), UK  Aune (2009), Uruguay  Williams (2010), USA  Hu (2011), Canada  Joshi (2015), USA  Miller (2013), USA | x  x  x  x  x  x  x  x  x  x  x |  |
| Cohort studies |  |  |
| Bostick (1994), USA  English (2004), Australia  Chao (2005), USA  Larsson (2005), Sweden  Norat (2005), Europe  Sato (2006), Japan  Cross (2007), USA  Lee (2009), China  Takachi (2011), Japan  Bernstein (2015), USA  Gilsing (2015), Netherlands  Oba (2006), Japan | x  x  x  x  x  x  x  x  x  x  x  x |  |

| **Rectal cancer** |  |  |
| --- | --- | --- |
| **Primary research articles included in review articles** | **Included systematic reviews** | |
| **First author, year, and country** | Zhao (2017) | |
| Case-control studies |  |  |
| Le Marchand (2002), USA  Murtaugh (2004), USA  Kimura (2007), Japan  Spencer (2010), UK  Aune (2009), Uruguay  Williams (2009), USA  Hu (2011), Canada  Joshi (2015), USA  Miller (2013), USA | x  x  x  x  x  x  x  x  x |  |
| Cohort studies |  |  |
| English (2004), Australia  Chao (2005), USA  Larsson (2005), Sweden  Norat (2005), Europe  Sato (2006), Japan  Cross (2007), Usa  Lee (2009), China  Takachi (2011), Japan  Bernstein (2015), USA  Gilsing (2015), Netherlands | x  x  x  x  x  x  x  x  x  x |  |

| **Cancer mortality** |  |  |
| --- | --- | --- |
| **Primary research articles included in review articles** | **Included systematic reviews** | |
| **First author, year, and country** | O´Sulivan (2013) | Wang (2016) |
| Cohort studies |  |  |
| Kappeler (2013), USA |  | x |
| Pan (2012), USA | x | x |
| Rohrmann (2013), Europe |  | x |
| Sinha (2009), USA | x | x |
| Whiteman (1999), UK | x | x |

| **Diabetes Mellitus** |  |
| --- | --- |
| **Primary research articles included in review articles** | **Included systematic reviews** |
| **First author, year, and country** | Micha (2010) |
| Cohort studies |  |
| Fung (2004), USA | x |
| Kröger (2009) (unpublished data), Germany | x |
| Meyer (2001), USA | x |
| Schulze (2003), USA | x |
| Song (2004), USA | x |
| Van Dam (2002), USA | x |
| Villegas (2006), China | x |

| **Coronary heart disease** |  |
| --- | --- |
| **Primary research articles included in review articles** | **Included systematic reviews** |
| **First author, year, and country** | Micha (2010) |
| Case-control studies |  |
| Martinez-Gonzalez (2002), Spain | x |
| Cohort studies |  |
| Burke (2007), Australia | x |
| Liu (2003), USA | x |
| Sinha (2009), USA | x |
| Whiteman (1999), UK | x |

| **Stroke** |  | |  |
| --- | --- | --- | --- |
| **Primary research articles included in review articles** | **Included systematic reviews** | | |
| **First author, year, and country** | Micha (2010) | Kim (2017) | |
| Cohort studies |  |  | |
| Fung (2004), USA | x |  | |
| Sauvaget (2003), Japan | x |  | |
| Bernstein (2012), USA |  | x | |
| Larsson (2011), Sweden |  | x | |
| Larsson (2011), Sweden |  | x | |
| Haring (2015), USA |  | x | |
| Amiano (2016), Spain |  | x | |

| **CVD mortality** |  |  |
| --- | --- | --- |
| **Primary research articles included in review articles** | **Included systematic reviews** | |
| **First author, year, and country** | O´Sulivan (2013) | Wang (2016) |
| Cohort studies |  |  |
| Kappeler (2013), USA |  | x |
| Nagao (2012), Japan |  | x |
| Pan (2012), USA | x | x |
| Rohrmann (2013), Europe |  | x |
| Sauvaget (2003), Japan | x |  |
| Sinha (2009), USA | x | x |
| Whiteman (1999), UK | x | x |
