# Supplementary material for: Processed meat intake and chronic disease morbidity and mortality: An overview of systematic reviews and meta-analyses
Source: PLoS One. 2019 Oct 17;14(10):e0223883. doi: 10.1371/journal.pone.0223883 (PMC6797176; doi:10.1371/journal.pone.0223883)
Supplement: S5 Table — (DOCX) [file pone.0223883.s005.docx]

**Supplemental Table 5**. Subgroup analyses relevant to this SR

|  | | **No. of SR** | **Results**  **RR (95% CI)** |
| --- | --- | --- | --- |
| ***Esophageal cancer*** | |  |  |
| **Study design** | |  |  |
| Case-control | 2 | **1.36 (1.07, 1.74)** (Choi, 2013)  **1.39 (1.00, 1.93)** (Zhu, 2014) |  |
| Cohort | 2 | 1.25 (0.83, 1.86) (Choi, 2013)  1.25 (0.83, 1.86) (Zhu, 2014) |  |
| **Dose** | | 1 | Dose-response increase of 100g/d:  1.37 (0.88, 2.13) (Choi, 2013) |
| **Meat species** | | - | - |
| **Method of preparation** | | - | - |
| **Demography** | |  |  |
| Male | 1 | 1.24 (0.58, 2.65) (Choi, 2013) |  |
| Female | 1 | 0.61 (0.33, 1.13) (Choi, 2013) |  |
| **Geography** | |  |  |
| Asia | 2 | 1.09 (0.61, 1.95) (Choi, 2013)  1.00 (0.52, 1.94) (Zhu, 2014) |  |
| Europe | 2 | 1.49 (0.99, 2.23) (Choi, 2013)  1.57 (0.95, 2.58) (Zhu, 2014) |  |
| United States | 2 | **1.30 (1.08, 1.57)** (Choi, 2013)  **1.23 (1.00, 1.50)** (Zhu, 2014) |  |
| South America | 2 | 0.76 (0.51, 1.13) (Choi, 2013)  1.07 (0.48, 2.41) (Zhu, 2014) |  |
| **Study quality** | |  |  |
| Newcastle-Ottawa Scale | 2 | ≥7: 1.20 (0.88, 1.62) (Choi, 2013)  <7: **1.43 (1.11, 1.86)** (Choi, 2013)  ≥7: **1.35 (1.03, 1.78)** (Zhu, 2014) |  |
|  | |  |  |
| ***Nasopharyngeal carcinoma*** | |  |  |
| **Study design** | | - |  |
| **Dose** | | 1 | < 30 g/wk vs. never: **1.46 (1.31, 1.64)** (Li, 2016)  30-60 g/week vs. never: **1.59 (1.33, 1.90)** (Li, 2016)  > 60 g/week vs. never: **2.11 (1.31, 3.42)** (Li, 2016) |
| **Meat species** | | - | - |
| **Method of preparation** | | - | - |
| **Demography** | | - | - |
| **Geography** | | - | - |
| **Study quality** | | - | - |

**Supplemental Table 5**. Subgroup analyses relevant to this SR **(continued)**

|  | | **No. of SR** | **Results**  **RR (95% CI)** | |
| --- | --- | --- | --- | --- |
|  | |  | **Case-control studies** | **Cohort studies** |
| ***Pancreatic cancer*** | |  |  |  |
| **Study design** | |  |  |  |
| **Dose** | | 1 | - | Dose-response for 50g/d increase:  Not reported (P=0.90) (Zhao, 2017) |
| **Meat species** | | - | - | - |
| **Method of preparation** | | - | - | - |
| **Demography** | |  |  |  |
| Male | 1 | - | **1.18 (1.06, 1.31)** (Zhao, 2017) |  |
| Female | 1 | - | 0.99 (0.84, 1.16) (Zhao, 2017) |  |
| **Geography** | |  |  |  |
| Asia | 1 | 3.21 (0.64, 16.10) (Zhao, 2017) | 1.16 (0.67, 2.01) (Zhao, 2017) |  |
| Europe | 1 | **1.32 (1.02, 1.70**) (Zhao, 2017) | 0.88 (0.74, 1.04) (Zhao, 2017) |  |
| America | 1 | 1.95 (0.95, 4.01) (Zhao, 2017) | **1.17 (1.01, 1.36)** (Zhao, 2017) |  |
| **Study quality** | |  |  |  |
| Newcastle-Ottawa Scale | 1 | ≥7: **1.80 (1.30, 2.49)** (Zhao, 2017)  <7: **1.61 (1.03, 2.51)** (Zhao, 2017) | >7: 1.08 (0.96, 1.22) (Zhao, 2017)  <7: 1.50 (0.60, 3.75) (Zhao, 2017) |  |
|  | |  |  |  |

**Supplemental Table 5**. Subgroup analyses relevant to this SR **(continued)**

|  | | **No. of SR** | **Results**  **RR (95% CI)** |
| --- | --- | --- | --- |
| ***Gastric cancer*** | |  |  |
| **Study design** | |  |  |
| Case-control | 1 | **1.64 (1.47, 1.83)** (Zhu, 2013) |  |
| Cohort | 1 | **1.18 (1.00, 1.38)** (Zhu, 2013) |  |
| **Dose** | | 2 | Processed meat:  Dose-response (per 1/week): 1.03 (0.99, 1.06) (Fang, 2015)  Dose-response for 50g/day increase: 1.21 (1.04, 1.41) (Zhao, 2017)  Ham-bacon-sausage:  Dose-response (per 1/week): 1.00 (0.98, 1.02) (Fang, 2015) |
| **Meat species** | | - | - |
| **Method of preparation** | | - | - |
| **Demography** | |  |  |
| Male | 1 | **1.26 (1.09, 1.46)** (Zhu, 2013) |  |
| Female | 1 | 1.16 (0.99, 1.36) (Zhu, 2013) |  |
| **Geography** | |  |  |
| Asia | 1 | **1.58 (1.06, 2.37)** (Zhu, 2013) |  |
| Europe | 2 | **1.50 (1.18, 1.91)** (Zhu, 2013)  **1.39 (1.15, 1.68)** (Fang, 2015) |  |
| United States | 2 | **1.17 (1.06, 1.29)** (Zhu, 2013)  1.05 (0.93, 1.19) (Fang, 2015) |  |
| South America | 1 | **1.94 (1.49, 2.52)** (Zhu, 2013) |  |
| **Study quality** | |  |  |
| Newcastle-Ottawa Scale | 1 | ≥7: **1.26 (1.10, 1.46)** (Zhu, 2013) |  |
|  | |  |  |
| ***Glioma*** | |  |  |
| **Study design** | |  |  |
| Case-control | 1 | Population-based: **1.26 (1.05, 1.51)** (Saneei, 2015)  Hospital-based: 0.79 (0.65, 0.97) (Saneei, 2015) |  |
| Cohort | 1 | 1.08 (0.84, 1.37) (Saneei, 2015) |  |
| **Dose** | | - | - |
| **Meat species** | | - | - |
| **Method of preparation** | | - | - |
| **Demography** | | - | - |
| **Geography** | | - | - |
| **Study quality** | |  |  |
| Newcastle-Ottawa Scale | 1 | <7: 1.25 (0.85, 1.84) (Saneei, 2015)  ≥7: 1.07 (0.94, 1.22) (Saneei, 2015) |  |
|  | |  |  |
| ***Ovarian cancer*** | |  |  |
| **Study design** | | - |  |
| **Dose** | | 1 | Increment of (4x30 g)/week: 1.07 (0.97, 1.17) (Wallin, 2011) |
| **Meat species** | | - | - |
| **Method of preparation** | | - | - |
| **Demography** | | - | - |
| **Geography** | | - | - |
| **Study quality** | |  |  |

**Supplemental Table 5**. Subgroup analyses relevant to this SR **(continued)**

|  | | **No. of SR** | **Results**  **RR (95% CI)** |
| --- | --- | --- | --- |
| ***Non-Hodgkin lymphoma*** | |  |  |
| **Study design** | |  |  |
| Case-control | 2 | **1.14 (1.00, 1.30)** (Solimini, 2016)  **1.20 (1.07, 1.36)** (Yang, 2015) |  |
| Cohort | 2 | - 1. (0.91, 1.11) (Solimini, 2016)   1.07 (0.96, 1.19) (Yang, 2015) |  |
| **Dose** | | - | - |
| **Meat species** | | - | - |
| **Method of preparation** | | - | - |
| **Demography** | |  |  |
| Male | 1 | 1.50 (0.98, 2.31) (Yang, 2015) |  |
| Female | 1 | 1.00 (0.69, 1.45) (Yang, 2015) |  |
| **Geography** | |  |  |
| Asia | - | - |  |
| Europe | 2 | 1.07 (0.85, 1.34) (Solimini, 2016)  **1.18 (1.02, 1.36)** (Yang, 2015) |  |
| North America | 2 | 1.12 (0.97, 1.29) (Solimini, 2016)  **1.19 (1.04, 1.36)** (Yang, 2015) |  |
| South America | 2 | 1.00 (0.77, 1.29) (Yang, 2015) |  |
| **Study quality** | |  |  |
| Newcastle-Ottawa Scale | 1 | <7: 1.79 (0.80, 3.98) (Yang, 2015)  ≥7: **1.15 (1.04, 1.26)** (Yang, 2015) |  |
|  | |  |  |
| ***Lung cancer*** | |  |  |
| **Study design** | |  |  |
| Case-control | 1 | 1.05 (0.75, 1.49) (Yang, 2012) |  |
| Cohort | 1 | 1.05 (0.92, 1.19) (Yang, 2012) |  |
| **Dose** | | - | - |
| **Meat species** | | - | - |
| **Method of preparation** | | - | - |
| **Demography** | |  |  |
| Male | 1 | 1.13 (0.85, 1.49) (Yang, 2012) |  |
| Female | 1 | 0.95 (0.84, 1.07) (Yang, 2012) |  |
| **Geography** | | - | - |
| **Study quality** | |  |  |
| Newcastle-Ottawa Scale | 1 | ≥7: 1.07 (0.90, 1.27) (Yang, 2012) |  |
|  | |  |  |
| ***Oral cavity and oropharynx cancer*** | |  |  |
| **Study design** | | - |  |
| **Dose** | | - | - |
| **Meat species** | | - | - |
| **Method of preparation** | | - | - |
| **Demography** | | 1 |  |
| **Geography** | |  |  |
| Asia | 1 | 2.09 (0.70, 6.29) (Xu, 2014) |  |
| Europe | 1 | 1.64 (0.59, 4.60) (Xu, 2014) |  |
| North America | - | - |  |
| South America | - | **1.93 (1.25, 3.00)** (Yang, 2015) |  |
| **Study quality** | | - | - |

**Supplemental Table 5**. Subgroup analyses relevant to this SR **(continued)**

|  | | **No. of SR** | **Results**  **RR (95% CI)** |
| --- | --- | --- | --- |
| ***Renal cell carcinoma*** | |  |  |
| **Study design** | |  |  |
| Case-control | 1 | **1.13 (1.00, 1.27)** (Zhang, 2017) |  |
| Cohort | 1 | 1.11 (0.99, 1.25) (Zhang, 2017) |  |
| **Dose** | | - | - |
| **Meat species** | | - | - |
| **Method of preparation** | | - | - |
| **Demography** | |  |  |
| Male | 1 | 1.50 (0.98, 2.31) (Yang, 2015) |  |
| Female | 1 | 1.00 (0.69, 1.45) (Yang, 2015) |  |
| **Geography** | |  |  |
| Asia | 1 | 1.60 (0.58, 4.44) (Zhang, 2017) |  |
| Europe | 1 | 0.98 (0.78, 1.23) (Zhang, 2017) |  |
| USA | 1 | **1.20 (1.07, 1.34)** (Zhang, 2017) |  |
| South America | 1 | 1.16 (0.81, 1.67) (Zhang, 2017) |  |
| **Study quality** | |  |  |
| Newcastle-Ottawa Scale | 1 | >6: 1.13 (1.03, 1.25) (Zhang, 2017)  ≤6: 1.03 (0.66, 1.63) (Zhang, 2017) |  |
|  | |  |  |
| ***Colorectal cancer*** | |  | (only for cohort studies) |
| **Study design** | | - | - |
| **Dose** | | 1 | 1.22 (1.12, 1.33) (Zhao, 2017) |
| **Meat species** | | - | - |
| **Method of preparation** | | - | - |
| **Demography** | | - | - |
| **Geography** | |  |  |
| Europe | | 1 | **1.26 (1.05, 1.51)** (Zhao, 2017) |
| America | | 1 | **1.12 (1.04, 1.21)** (Zhao, 2017) |
| Asia-Australia | | 1 | 1.17 (0.88, 1.56) (Zhao, 2017) |
| **Quality score** | |  |  |
| Newcastle-Ottawa Scale | | 1 | ≥7: **1.14 (1.07, 1.21)** (Zhao, 2017)  <7: 1.32 (0.89, 1.94) (Zhao, 2017) |
|  | |  |  |
| ***Colon cancer*** | |  | (only for cohort studies) |
| **Study design** | | - | - |
| **Dose** | | 1 | **1.23 (1.11, 1.37)** (Zhao, 2017) |
| **Meat species** | | - | - |
| **Method of preparation** | | - | - |
| **Demography** | | - | - |
| **Geography** | |  |  |
| Europe | | 1 | **1.27 (1.01, 1.59)** (Zhao, 2017) |
| America | | 1 | **1.20 (1.10, 1.31)** (Zhao, 2017) |
| Asia-Australia | | 1 | 1.23 (0.95, 1.59) (Zhao, 2017) |
| **Quality score** | |  |  |
| Newcastle-Ottawa Scale | | 1 | ≥7: **1.22 (1.13, 1.32)** (Zhao, 2017)  <7: 1.02 (0.60, 1.74) (Zhao, 2017) |
|  | |  |  |
| ***Rectal cancer*** | |  |  |
| **Study design** | | - | - |
| **Dose** | | 1 | 1.22 (0.99, 1.28) (Zhao, 2017) |
| **Meat species** | | - | - |
| **Method of preparation** | | - | - |
| **Demography** | | - | - |

**Supplemental Table 5**. Subgroup analyses relevant to this SR **(continued)**

|  | | **No. of SR** | **Results**  **RR (95% CI)** |
| --- | --- | --- | --- |
| ***Rectal cancer (continued)*** | |  |  |
| **Geography** | |  |  |
| Europe | | 1 | **1.23 (1.06, 1.42)** (Zhao, 2017) |
| America | | 1 | 1.34 (0.84, 2.13) (Zhao, 2017) |
| Asia-Australia | | 1 | 1.04 (0.69, 1.57) (Zhao, 2017) |
| **Quality score** | |  |  |
| Newcastle-Ottawa Scale | | 1 | ≥7: 1.13 (0.95, 1.35) (Zhao, 2017)  <7: 1.49 (0.83, 2.67) (Zhao, 2017) |
|  | |  |  |
| ***Cancer mortality*** | |  |  |
| **Study design** | |  | - |
| **Dose** | | - | - |
| **Meat species** | | - | - |
| **Method of preparation** | | - | - |
| **Demography** | |  |  |
| Male | 1 | **1.10 (1.03, 1.18)** (Wang, 2016) |  |
| Female | 1 | **1.10 (1.06, 1.15)** (Wang, 2016) |  |
| **Geography** | |  |  |
| Asia | - | - |  |
| Europe | 1 | **1.06 (1.02, 1.10)** (Wang, 2016) |  |
| North America | 1 | **1.09 (1.06, 1.12)** (Wang, 2016) |  |
| South America | - | - |  |
| **Study quality** | |  |  |
| Combination of MOOSE, QUATSO and STROBE (0,6) | 1 | High (4/5): **1.08 (1.06,1.11)** (Wang, 2016)  Low (<4): 0.98 (0.58, 1.67) (Wang, 2016) |  |
|  | |  |  |
| ***Stroke*** | |  |  |
| **Study design** | | - | - |
| **Dose** | | - | - |
| **Meat species** | | - | - |
| **Method of preparation** | | - | - |
| **Demography** | |  |  |
| Male | | 1 | 1.21 (1.09, 1.34) (Kim, 2017) |
| Female | | 1 | 1.12 (1.01, 1.24) (Kim, 2017) |
| **Geography** | | - | - |
| **Study quality** | | - | - |
|  | |  |  |
| ***CVD mortality*** | |  |  |
| **Study design** | |  | - |
| **Dose** | | - | - |
| **Meat species** | | - | - |
| **Method of preparation** | | - | - |
| **Demography** | |  |  |
| Male | 1 | 1.06 (0.92, 1.23) (Wang, 2016) |  |
| Female | 1 | **1.26 (1.19, 1.33)** (Wang, 2016) |  |
| **Geography** | |  |  |
| Asia | - | 0.22 (0.04, 1.15) (Wang, 2016) |  |
| Europe | 1 | **1.15 (1.09, 1.21)** (Wang, 2016) |  |
| North America | 1 | **1.16 (1.06, 1.27)** (Wang, 2016) |  |
| South America | - | - |  |
| **Study quality** | |  |  |
| Combination of MOOSE, QUATSO and STROBE (0,6) | 1 | High (4/5): **1.15 (1.07,1.24)** (Wang, 2016)  Low (<4): 1.27 (0.36, 4.44) (Wang, 2016) |  |

|  | **No. of SR** | **Results**  **RR (95% CI)** |
| --- | --- | --- |
| ***Type II diabetes*** |  |  |
| Study design | - | (Cohort studies only) |
| Dose | - | - |
| Meat species | - | - |
| Method of preparation | - | - |
| Demography | - | - |
| Geography | - | - |
| Study quality | - | - |
|  |  |  |
| ***CVD*** |  |  |
| Study design | - | - |
| Dose | - | - |
| Meat species | - | - |
| Method of preparation | - | - |
| Demography | - | - |
| Geography | - | - |
| Study quality | - | - |
