# Supplementary material for: Processed meat intake and chronic disease morbidity and mortality: An overview of systematic reviews and meta-analyses
Source: PLoS One. 2019 Oct 17;14(10):e0223883. doi: 10.1371/journal.pone.0223883 (PMC6797176; doi:10.1371/journal.pone.0223883)
Supplement: S6 Table — (DOCX) [file pone.0223883.s006.docx]

**Cancers**

*Esophageal cancer*

The two systematic reviews that examined incidence of esophageal cancer as outcome were both graded with an AMSTAR score of 6 (25;39). Neither of the two systematic reviews provided an “a priori” design (AMSTAR item number 1), and did not include i.e. “grey literature” or “unpublished literature” as inclusion criterion (AMSTAR item number 4), or provided a list of excluded studies (AMSTAR item number 5). Furthermore, neither of the systematic reviews considered the scientific quality in formulating the conclusions (AMSTAR item number 8), nor provided a list of the potential conflict of interest of the authors of the included studies (AMSTAR item number 11; **Supplemental table 3**).

*Head and neck cancer (nasopharyngeal carcinoma)*

In relation to nasopharyngeal carcinoma, we included one systematic review (28), which was graded with an AMSTAR score of 5 in our quality assessment, as this systematic review did not provide an “a priori” design (AMSTAR item number 1), and did not specify if there was a duplicate study selection (AMSTAR item number 2). Furthermore, the authors did not include i.e. “grey literature” or “unpublished literature” as inclusion criterion (AMSTAR item number 4), and did not provide a list of the excluded studies (AMSTAR item number 5). Finally, this systematic review did not consider the scientific quality in formulating the conclusions (AMSTAR item number 8), and did not provide a list of the conflict of interest of authors of the included studies (AMSTAR item number 11; **Supplemental table 3**).

*Pancreatic cancer*

One systematic review examined epidemiologic evidence on the association between processed meat consumption and pancreatic cancer risk and was graded with an AMSTAR score of 5. This score was obtained because this systematic review did not provide an “a priori” design (AMSTAR item number 1) and it was unclear whether data extraction was performed in duplicate and what was the consensus procedure (AMSTAR item number 2). In addition, the authors did not include i.e. “grey literature” or “unpublished literature” as inclusion criterion (AMSTAR item number 4), and did not provide a list of the excluded studies (AMSTAR item number 5). Lastly, this systematic review did not consider the scientific quality when formulating the conclusions (AMSTAR item number 8), and did not provide a list of the conflict of interest of authors of the included studies (AMSTAR item number 11) (**Supplemental table 3**).

*Liver cancer (hepatocellular carcinoma)*

One systematic review that evaluated the relationship between processed meat intake and risk of hepatocellular carcinoma (29) we graded with an AMSTAR score of 5. The reason for the given score was that an “a priori” design was not provided (AMSTAR item number 1), and the authors did not specify whether there was a duplicate study selection (AMSTAR item number 2). Also, whether publication status was used as inclusion criteria was not stated (AMSTAR item number 4), and a list of excluded studies was not provided (AMSTAR item number 5). This systematic review did not consider the scientific quality in formulating the conclusions (AMSTAR item number 8), and did not provide a list of the conflict of interest of the authors of the included studies (AMSTAR item number 11; **Supplemental table 3**).

*Gastric cancer*

Three systematic reviews (23;26;38) and one systematic review of systematic reviews (27) were conducted with risk of gastric cancer as outcome. The AMSTAR scores were 6, 6 and 7 for the three systematic reviews (23;26;38), and 6 for the systematic review of systematic reviews. Neither Fang et al. (26), Li et al. (27), Zhao et al. (23) or Zhu et al. (38) provided an “a priori” design (AMSTAR item number 1). Fang et al. (26) , Zhao et al. (23) and Zhu et al. (38) did not include i.e. “grey literature” or “unpublished literature” as inclusion criterion (AMSTAR item number 4), did not consider the scientific quality in formulating the conclusions (AMSTAR item number 8), and did not provide a list of the conflict of interest of the authors of the included studies (AMSTAR item number 11). Neither Fang et al. (2015) (26), Li et al. (27) nor Zhao et al. (23) provided a list of excluded studies (AMSTAR item number 5). Finally, Li et al. (27) did not include the characteristics of the included studies (AMSTAR item number 6; **Supplemental table 3**).

*Brain cancer (glioma)*

We included one systematic review (32) of processed meat intake and risk of glioma, which received an AMSTAR score of 8, and one systematic review of systematic reviews (31) which received an AMSTAR score of 7. The reasons for the given score were that the authors of this review of reviews (31) did not include i.e. “grey literature” or “unpublished literature” as inclusion criterion (AMSTAR item number 4), did not consider the scientific quality when formulating the conclusions (AMSTAR item number 8), and did not provide a list of the conflict of interest of the authors of the included studies (AMSTAR item number 11; **Supplemental table 3**).

*Ovarian cancer*

We included one systematic review (34) that examined processed meat intake and risk of ovarian cancer, which we graded with an AMSTAR score of 6. The reason for the given score was that there was not provided an “a priori” design (AMSTAR item number 1), and the authors did not specify whether there was a duplicate study selection and data extraction (AMSTAR item number 1). Whether publication status was used as inclusion criteria was not stated either (AMSTAR item number 4), and the authors did not consider the scientific quality in formulating the conclusions (AMSTAR item number 8). Also, the authors did not provide a list of the conflict of interest of the authors of the included studies (**Supplemental table 3**).

*Non-Hodgkin lymphoma*

We included two systematic reviews on processed meat intake and risk of Non-Hodgkin lymphoma (33;37), both graded with the AMSTAR score of 7. Neither of the two reviews provided an “a priori” design (AMSTAR item number 1), or specified whether publication status was used as inclusion criteria (AMSTAR item number 4). Furthermore, they did not provide a list of the conflict of interest of the included studies (AMSTAR item number 11). Yang et al. (37) also did not report a list of excluded studies (AMSTAR item number 5), and Solimini et al. (33) did not consider the scientific quality in formulating the conclusions (AMSTAR item number 8) (**Supplemental table 3**).

*Lung cancer*

This systematic review (36) was graded with an AMSTAR score of 7, and included 6 case-control studies and 4 cohort studies. The reasons for the AMSTAR score were that no “a priori” design was provided (AMSTAR item number 1), and that the information on whether publication status was used as inclusion criteria was not reported (AMSTAR item number 4). No list of excluded studies (AMSTAR item number 5) or conflict of interest of the authors of the included studies was provided (AMSTAR item number 11; **Supplemental table 3**).

*Oral cavity and orophanx cancer*

The systematic review with risk of oral cavity and orophanx cancer as an outcome reached the AMSTAR score of 7 and was based on 10 case-control studies (35). The AMSTAR score of 7 reflected a lack of an “a priori” design (AMSTAR item number 1), no reporting on whether publication status was used as an inclusion criterion (AMSTAR item number 4), that the quality score was not used when formulating the conclusion (AMSTAR item number 8) and no list of conflict of interest of the authors of the included studies were provided (AMSTAR item number 11) (**Supplemental table 3**).

*Renal cell carcinoma*

One systematic review with risk of renal cell carcinoma as outcome was identified (21), which reached an AMSTAR score of 6. This score is justified by the fact that no “a priori” design was provided (AMSTAR item number 1); the authors did not include i.e. “grey literature” or “unpublished literature” as inclusion criterion (AMSTAR item number 4); did not provide a list of the excluded studies (AMSTAR item number 5); did not consider the scientific quality when formulating the conclusions (AMSTAR item number 8), and lastly did not provide a list of the conflict of interest of authors of the included studies (AMSTAR item number 11; **Supplemental table 3**).

*Colorectal cancer*

It was identified one systematic review that examined the evidence on the associations between processed meat and the risk of colorectal cancer (CRC) (22). This systematic review was graded with an AMSTAR score of 6, because the authors: did not provide an “a priori” design (AMSTAR item number 1); did not include i.e. “grey literature” or “unpublished literature” as inclusion criterion (AMSTAR item number 4); did not provide a list of the excluded studies (AMSTAR item number 5); did not consider the scientific quality when formulating the conclusions (AMSTAR item number 8), and lastly did not provide a list of the conflict of interest of authors of the included studies (AMSTAR item number 11; **Supplemental table 3**).

*Cancer mortality*

The conclusions from the two systematic reviews (12;30) included in the present review were that intake of processed meat is associated with approximately 10% increased risk of cancer mortality (**Table 3**). Both of the systematic reviews were graded with an AMSTAR score of 7. Neither of the systematic reviews provided an “a priori” design (AMSTAR item number 1), or stated whether publication status was used as inclusion criteria (AMSTAR item number 4); no lists of conflict of interest of the authors of the included studies were provided either (AMSTAR item number 11). O´Sulivan et al. (30) furthermore did not provide a list of excluded studies (AMSTAR item number 5), and Wang et al. (12) did not consider the scientific quality when formulating the conclusion (AMSTAR item number 8) (**Supplemental table 3**).

**Systematic reviews on diabetes**

We included one systematic review by Micha et al. that examined relations between processed meat intake and T2D risk (14). The AMSTAR score assigned to this study was 7. The review did not provide an “a priori” design (AMSTAR item number 1), and did not report whether the process of data selection and data extraction was duplicated (AMSTAR item number 2). The scientific quality was not considered in formulating the conclusion (AMSTAR item number 8), and no list of conflict of interest of the authors of the included studies were provided (AMSTAR item number 11) (**Supplemental table 3**).

**Systematic reviews on cardiovascular disease**

Regarding the risk of CVD, a total of 4 systematic reviews were identified, which presented in total results for the association between processed meat consumption and risk of coronary heart diseases (CHD) (14), risk of stroke (14;40) and CVD mortality (12;30). Kim et al.’s systematic review received an AMSTAR score of 6, while the 3 other (O’ Sulivan et al., Wang et al. and Micha et al.) received each an AMSTAR score of 7. The reason for the score given to O’ Sulivan et al., Wang et al. and Micha et al. is provided in the cancer mortality and diabetes section, since each of these systematic reviews included multiple outcomes and thus presented multiple results in the same paper (12;14;30). Kim et al. obtained an AMSTAR score of 6 because the authors: did not provide an “a priori” design (AMSTAR item number 1); did not include i.e. “grey literature” or “unpublished literature” as inclusion criterion (AMSTAR item number 4); did not provide a list of the excluded studies (AMSTAR item number 5); did not consider the scientific quality when formulating the conclusions (AMSTAR item number 8), and lastly did not provide a list of the conflict of interest of authors of the included studies (AMSTAR item number 11) (**Supplemental table 3**).

**Results from post hoc quality assessment analyses**

We performed a post hoc quality assessment of the one systematic review that was a priori not included because its search was performed in one database only (AMSTAR item number 3), but did assess quality (AMSTAR item number 7) (**Figure 1**) (completed by MNH and AS). The additional review had stroke as outcome and received an AMSTAR score of 6 (41) (**Supplemental table 3**).
